# Supplementary figures and images for: Polygenic adaptation of rosette growth in Arabidopsis thaliana
Source: PLoS Genet. 2021 Jan 25;17(1):e1008748. doi: 10.1371/journal.pgen.1008748 (PMC7861555; doi:10.1371/journal.pgen.1008748)

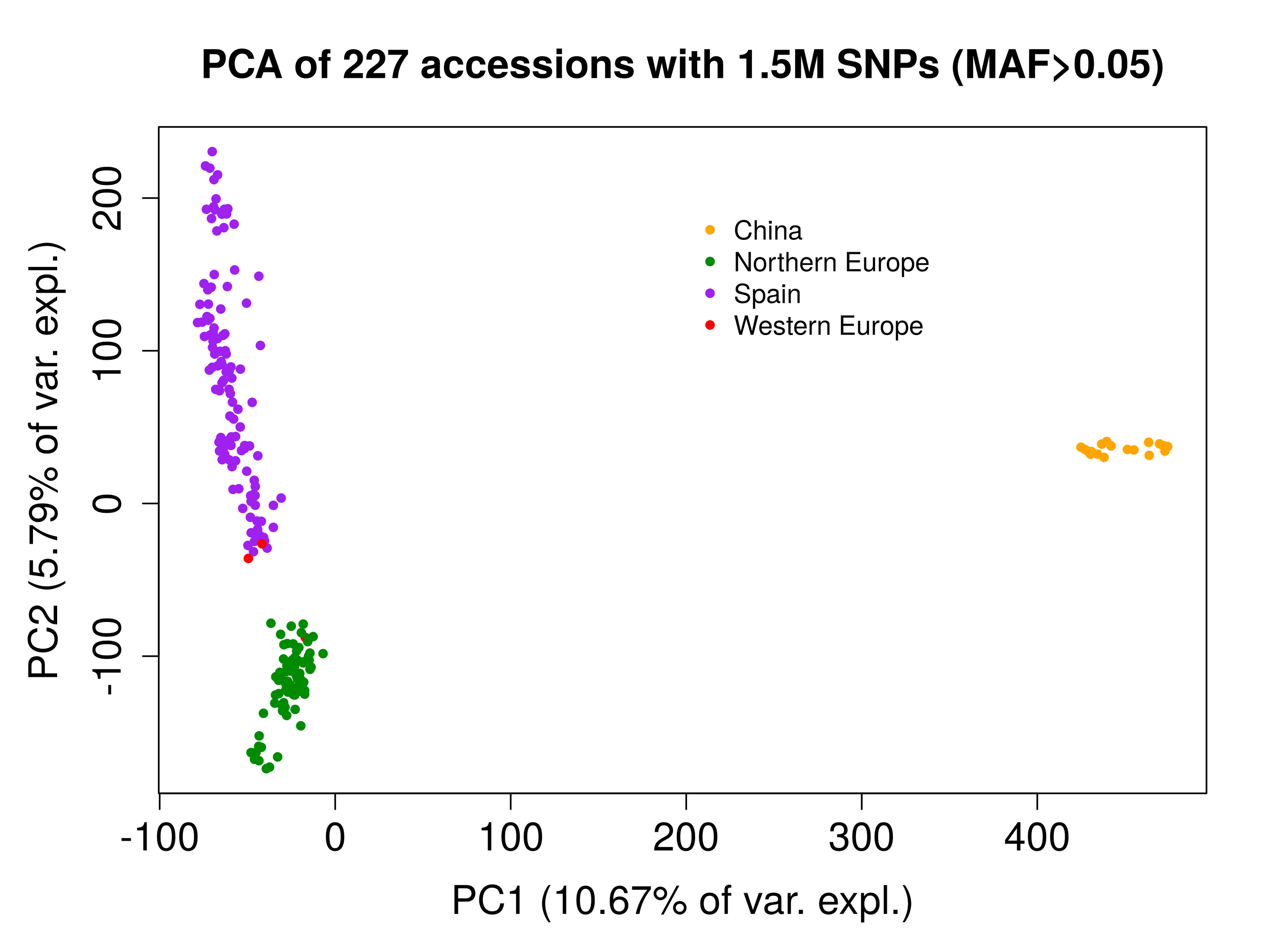

Supplement: S2 Fig — The PCA is based on 1.5 millions SNPs with a minor allele frequency larger than 0.05. The first two principle components explain about 16% of the variance between the genotypes. Regions: China (orange), Northern Europe (green), Spain (purple), Western Europe (red). (TIFF) [file pgen.1008748.s002.tiff]

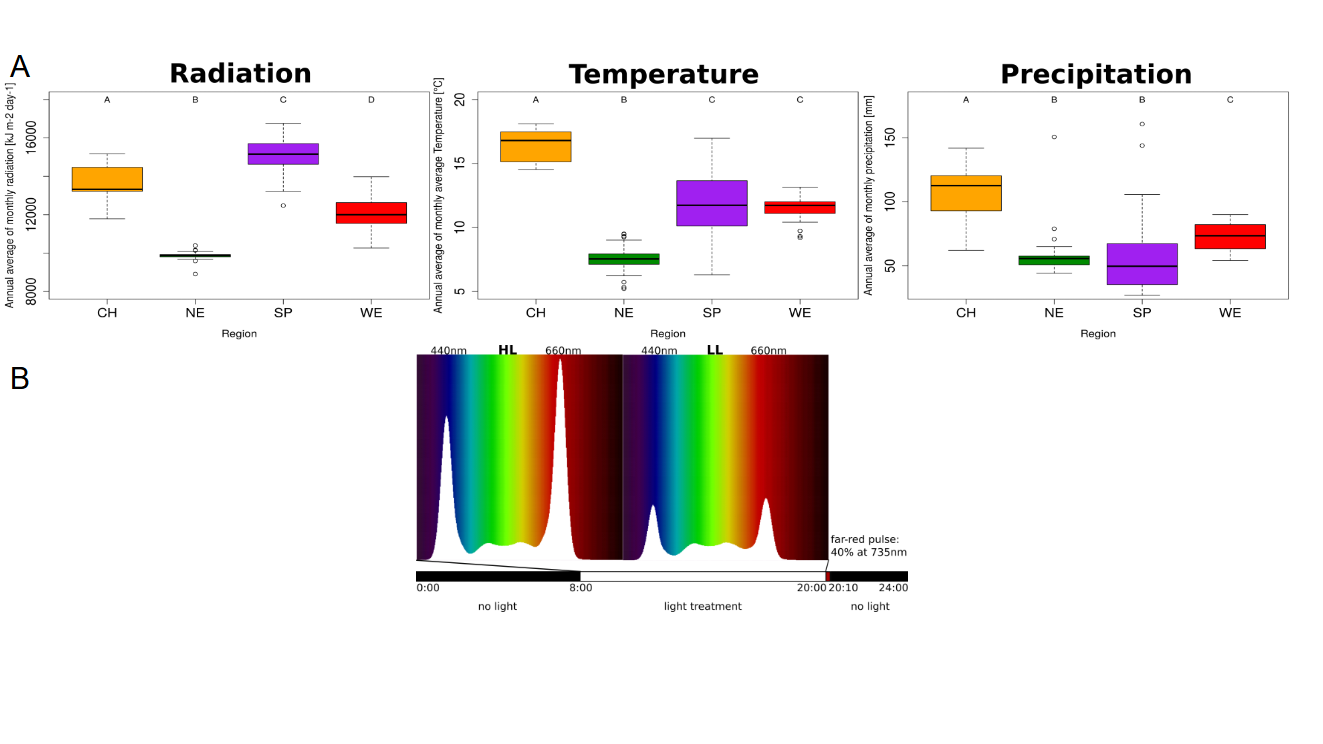

Supplement: S3 Fig — A) Annual average of the monthly radiation (left), monthly average temperature (center) and monthly precipitation (right) estimates for the sampling location of each genotype from Worldclim2 data (estimate per ~1km2). Boxplots with different letters are significantly different according to Tukey’s HSD (p-value < 0.05). Region information: China (CH, 20 unique locations), Northern Europe (NE, 46 unique locations), Spain (SP, 120 unique locations) & Western Europe (WE, 15 unique locations). B) Experimental Set-up in the growth chamber with the light-spectrum and intensity in HL (left) and LL (right). The bottom bar represent the timing of the light. (TIF) [file pgen.1008748.s003.tif]

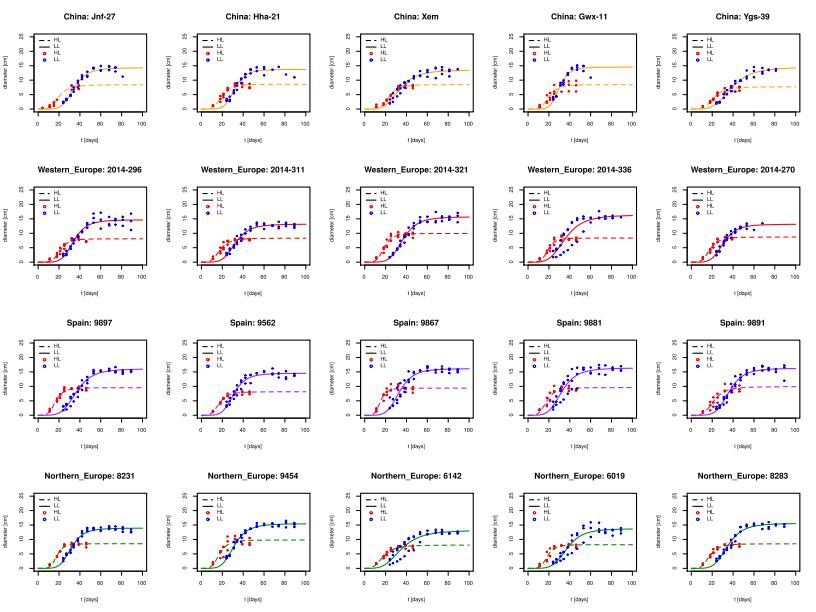

Supplement: S4 Fig — Predicted growth curves averaged per genotype (from drm function). To represent the regions 5 genotypes per region were chosen randomly. The growth curves were estimated from diameter measurements at different time points (points for three input replicates). Diameter measurements for HL are from day 11 to 46 and for LL from day 24 to 89. Legend: Title: Region and ID; HL (dashed line, red points), LL (solid line, blue points), China (orange), Northern Europe (green), Spain (purple) and Western Europe (red). (TIF) [file pgen.1008748.s004.tif]

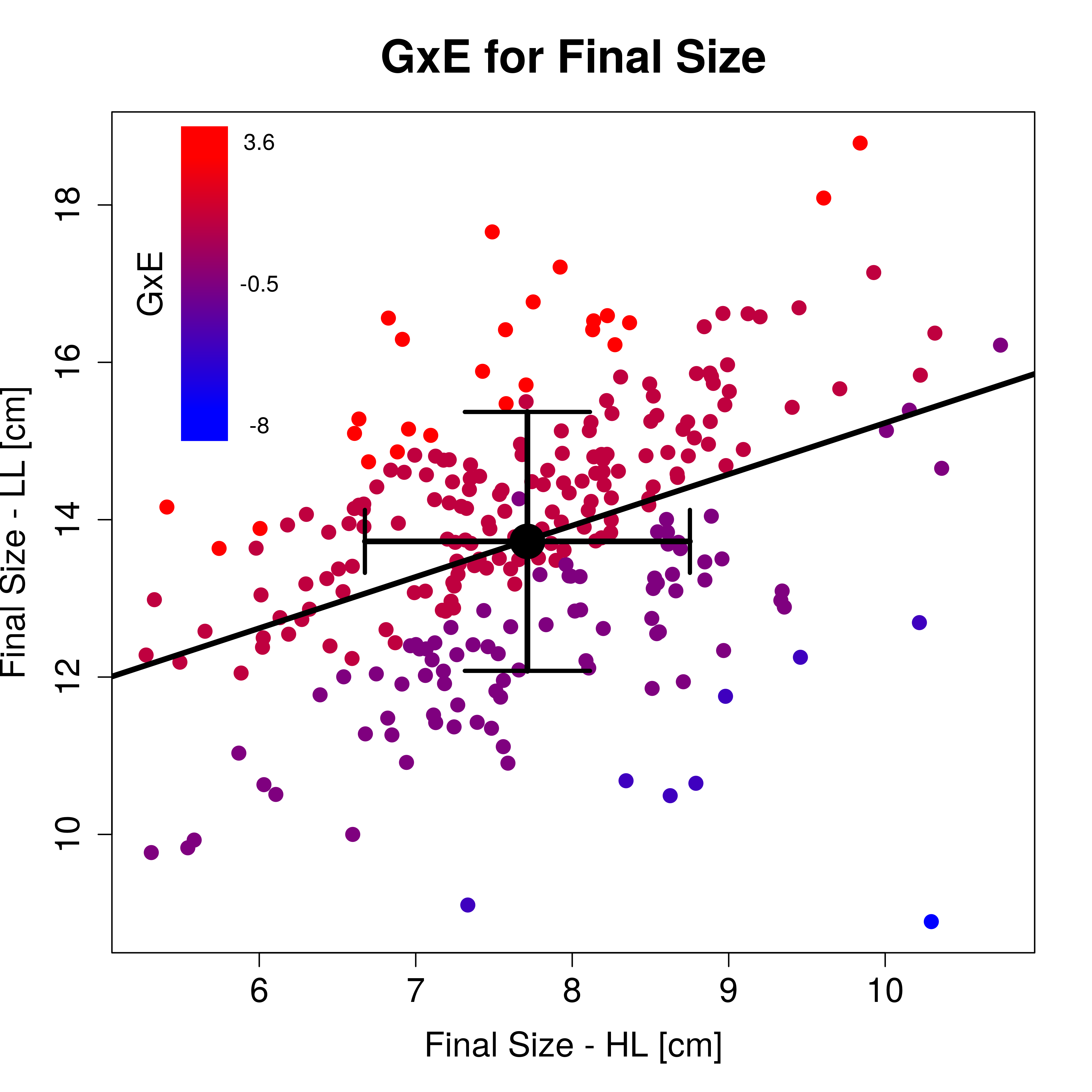

Supplement: S5 Fig — GxE was estimated based on a glm(Final Size ~ genotype * environment) and is indicated by the color. Each dot corresponds to a genotype with its phenotype in HL (x-axis) and LL (y-axis) (269 genotypes in total). The black dot shows the average over all genotypes with standard deviation. The line shows a linear model for Final Size in LL ~ Finals Size HL. (TIF) [file pgen.1008748.s005.tif]

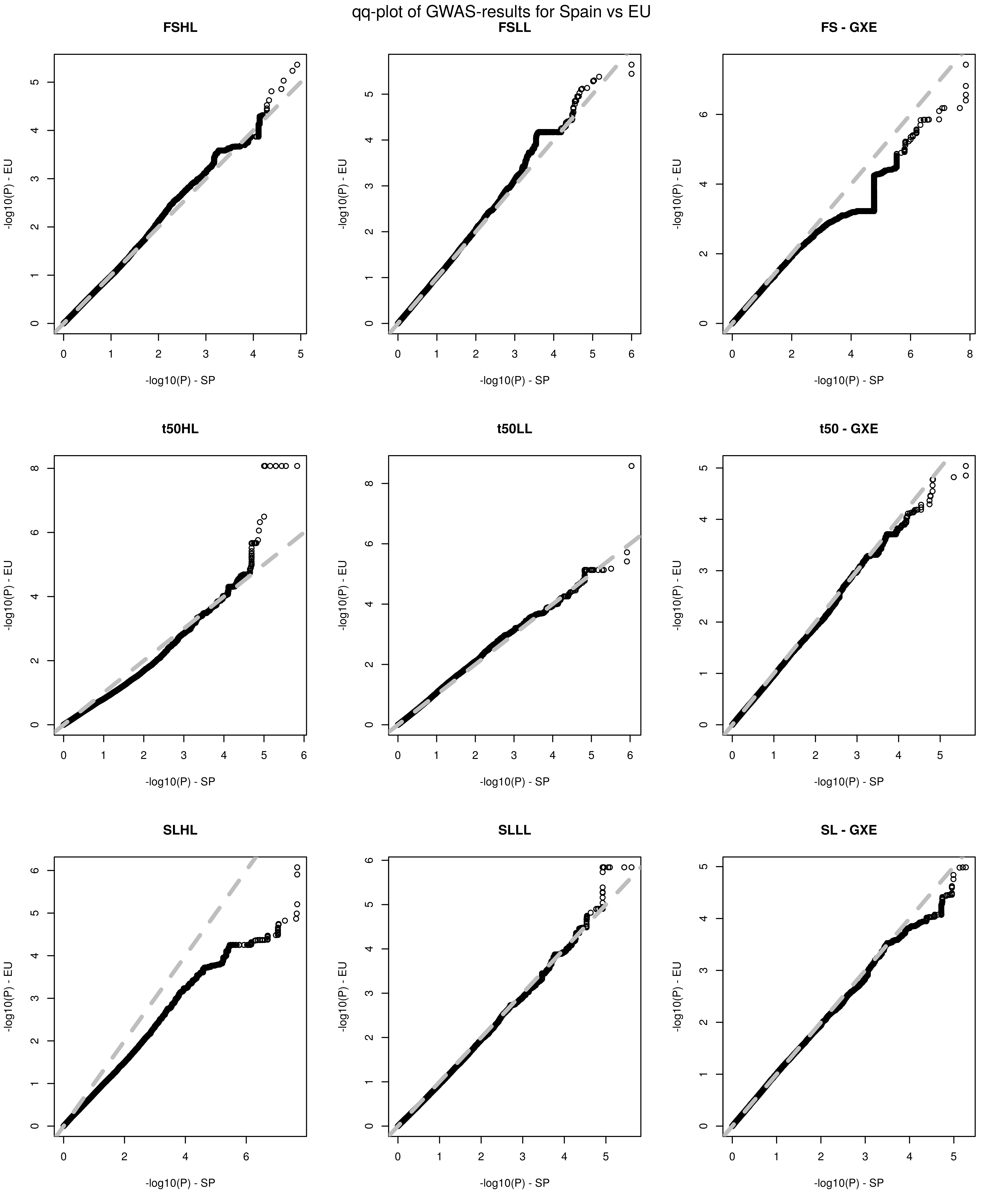

Supplement: S6 Fig — qq-plots comparing the GWAS data in S13 Fig (Spain, 117 genotypes, x-axis) to the data from S12 Fig (Europe, 201 genotypes, y-axis). The traits are Final Size (upper row), t50 (2nd row) and Slope (lower row) in HL (left column), LL (middle column) and their GxE (right column). The grey dotted line indicates the neutral expectation. (TIF) [file pgen.1008748.s006.tif]

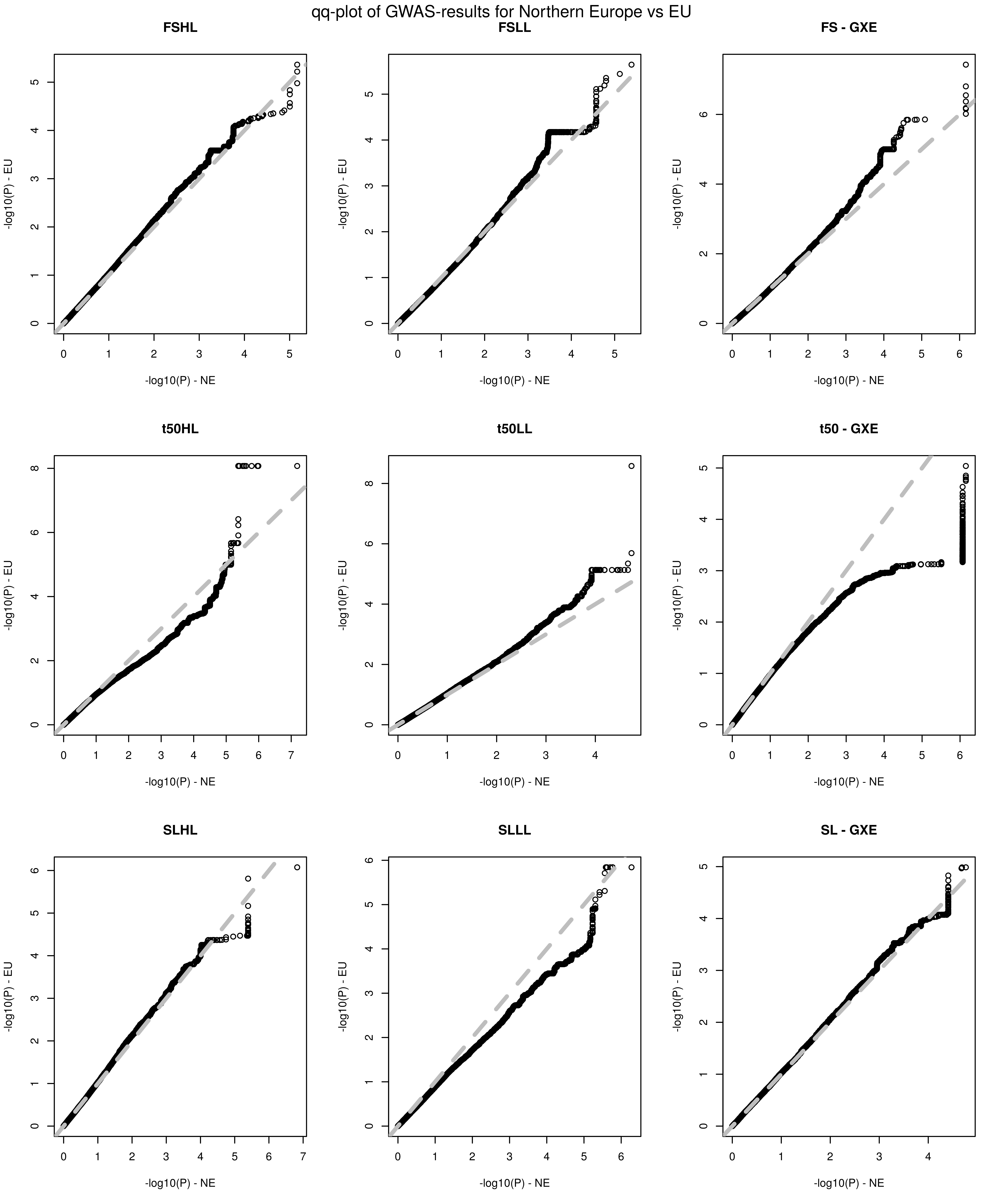

Supplement: S7 Fig — qq-plots comparing the GWAS data in S14 Fig (Northern Europe, 83 genotypes, x-axis) to the data from S12 Fig (Europe, 201 genotypes, y-axis). The traits are Final Size (upper row), t50 (2nd row) and Slope (lower row) in HL (left column), LL (middle column) and their GxE (right column). The grey dotted line indicates the neutral expectation. (TIF) [file pgen.1008748.s007.tif]

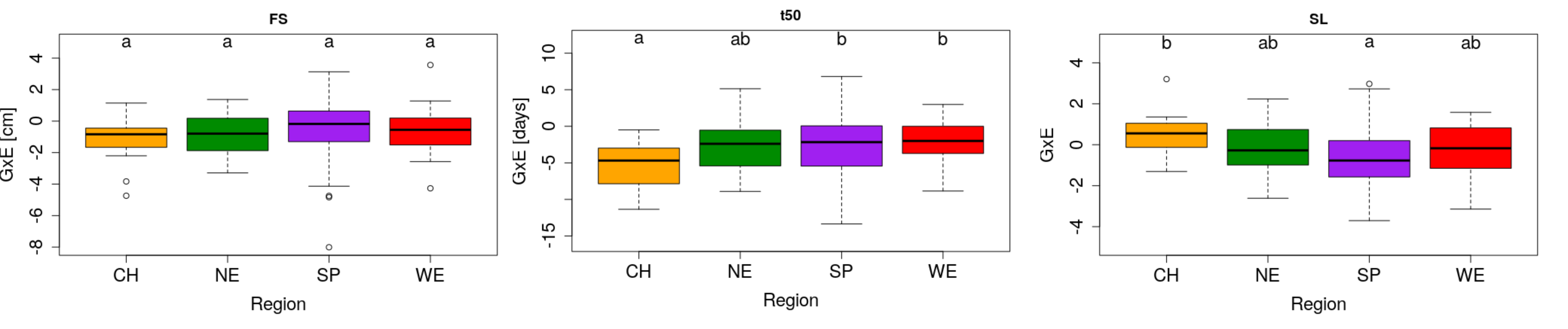

Supplement: S8 Fig — GxE for FS (left), t50 (center) and Slope (SL, right). The phenotypic values are based on 217 genotypes of Arabidopsis thaliana. Groups that do not share a letter are significantly different according to Tukey’s HSD (p-value < 0.05). Region information: China (CH, n = 14), Northern Europe (NE, n = 58), Spain (SP, n = 117) & Western Europe (WE, n = 28). (TIF) [file pgen.1008748.s008.tif]

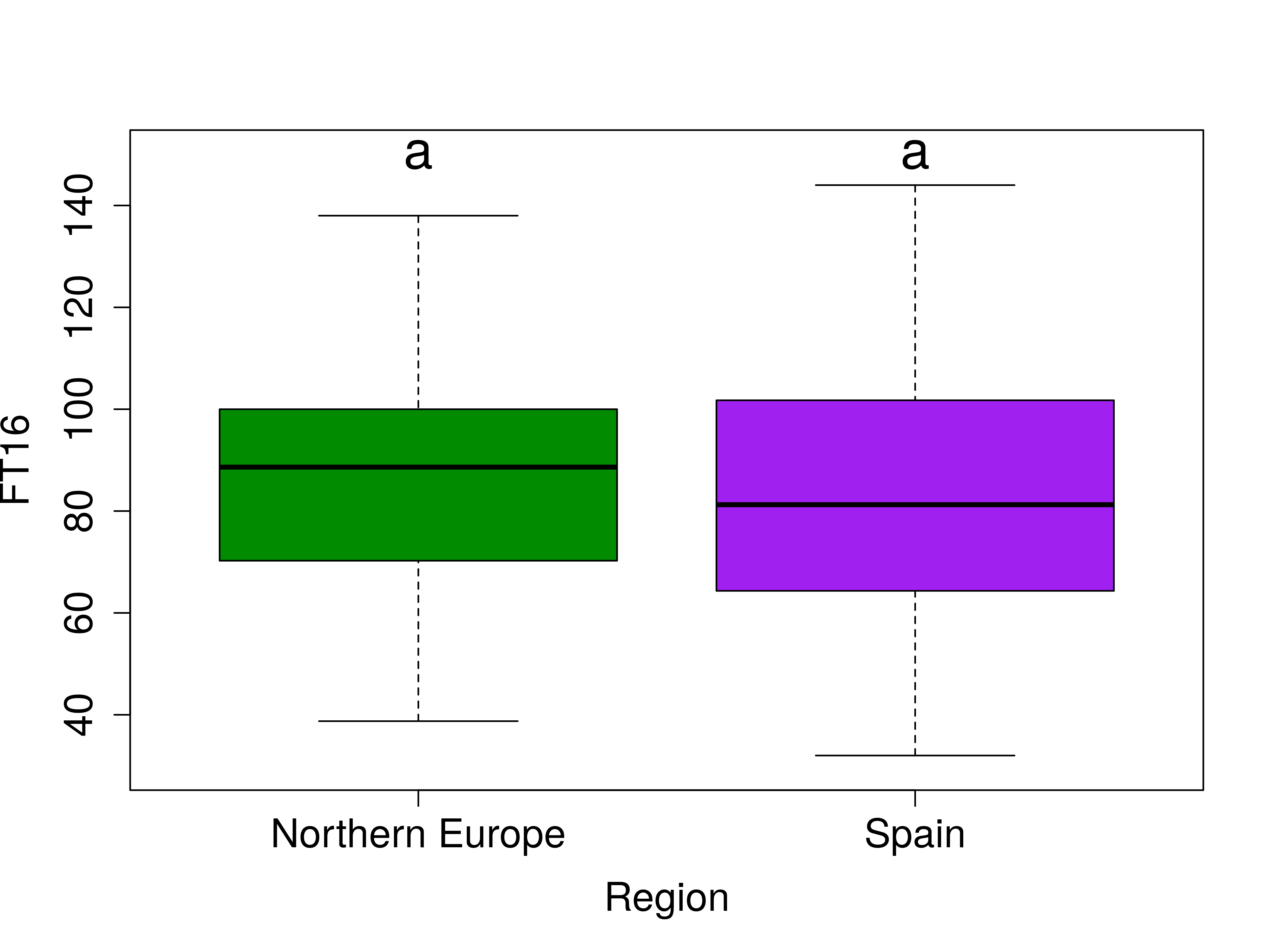

Supplement: S9 Fig — Flowering time in 16°C conditions of each genotype plotted for Northern Europe (green, n =) and Spain (purple, n =, based on data from 1001Genomes, 2016). The regions showed no phenotypic difference, as indicated by the same letter (pairwise GLHT, p-value> 0.05). (TIF) [file pgen.1008748.s009.tif]

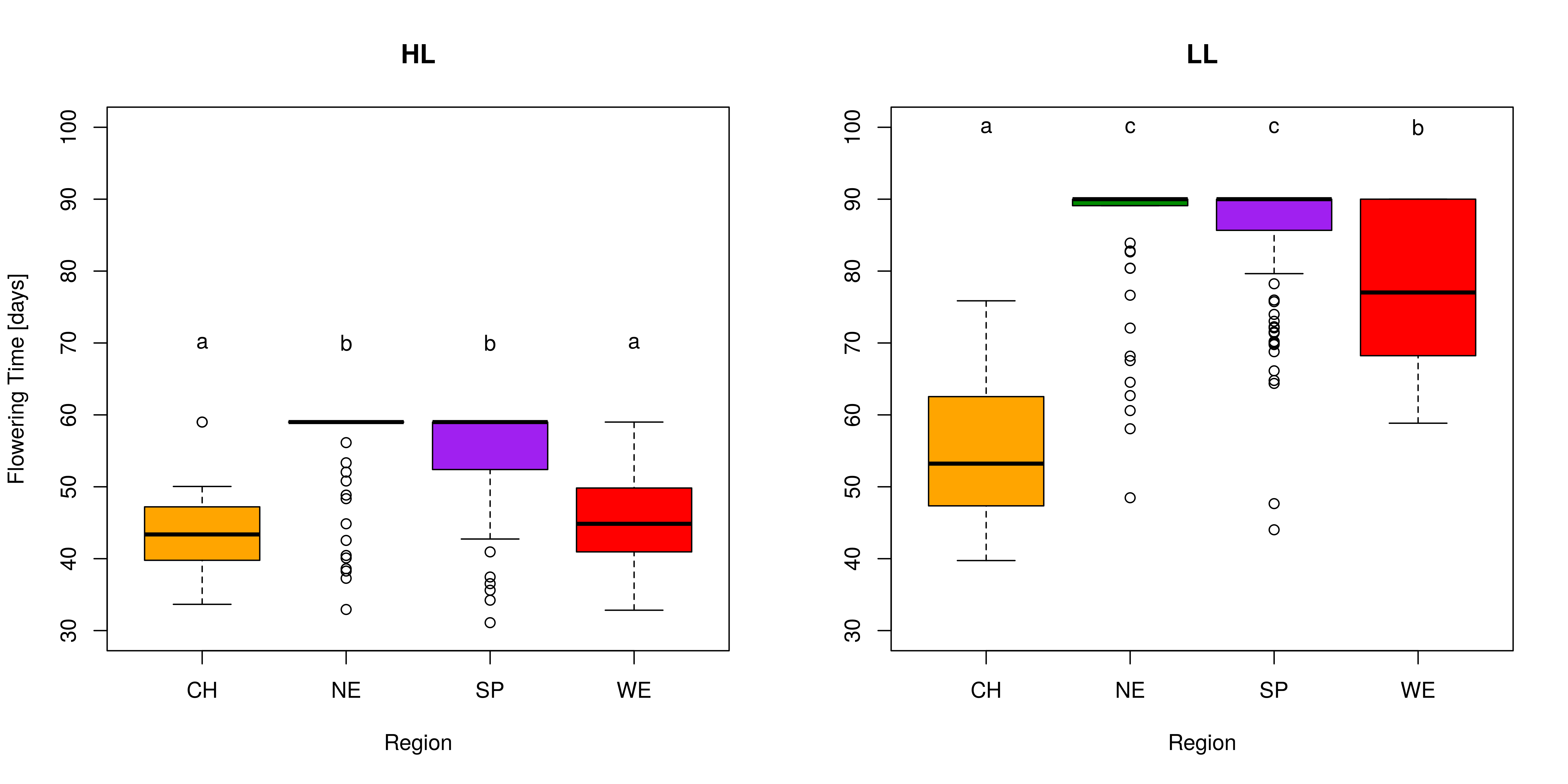

Supplement: S10 Fig — Flowering time of each genotype in HL (left) and LL conditions(right). Missing values were replaced with 59 (HL) or 90 (LL) days after sowing. Boxplots with different letters are significantly different according to Tukey’s HSD (p-value < 0.05). Population information: China (CH, n = 22), Northern Europe (NE, n = 84), Spain (SP, n = 121) & Western Europe (WE, n = 53). (TIF) [file pgen.1008748.s010.tif]

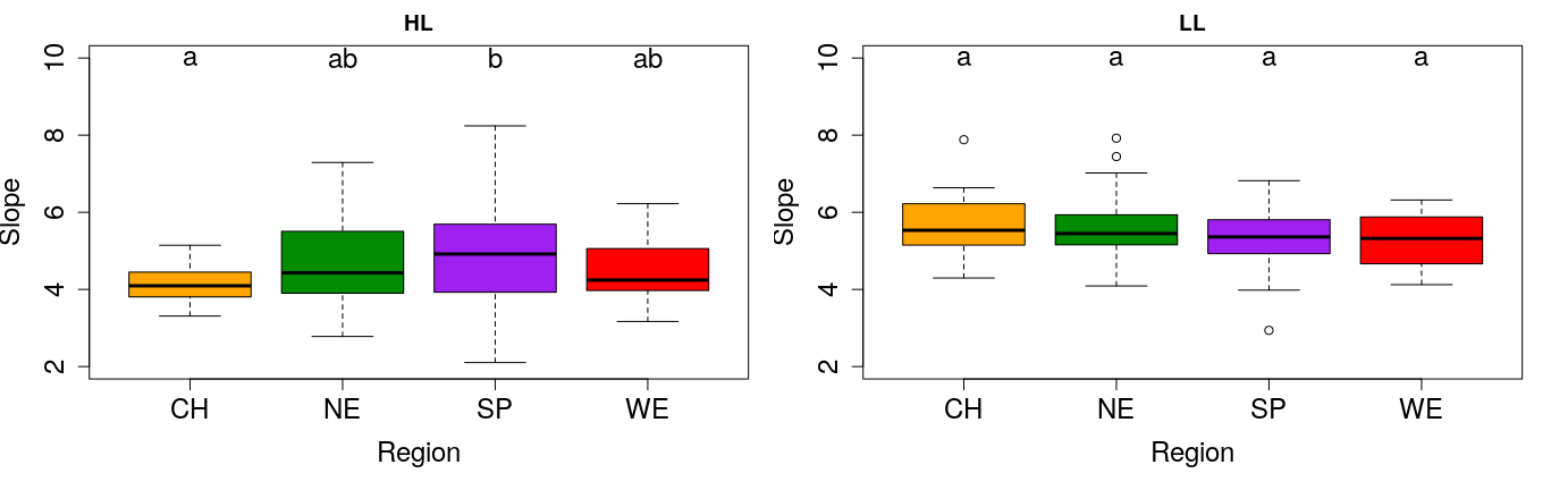

Supplement: S11 Fig — The phenotypic values are based on 220 genotypes of Arabidopsis thaliana in HL (left) and LL conditions (right). Groups that do not share a letter are significantly different according to Tukey’s HSD (p-value < 0.05). Region information: China (CH, n = 15), Northern Europe (NE, n = 58), Spain (SP, n = 119) & Western Europe (WE, n = 28). (TIF) [file pgen.1008748.s011.tif]

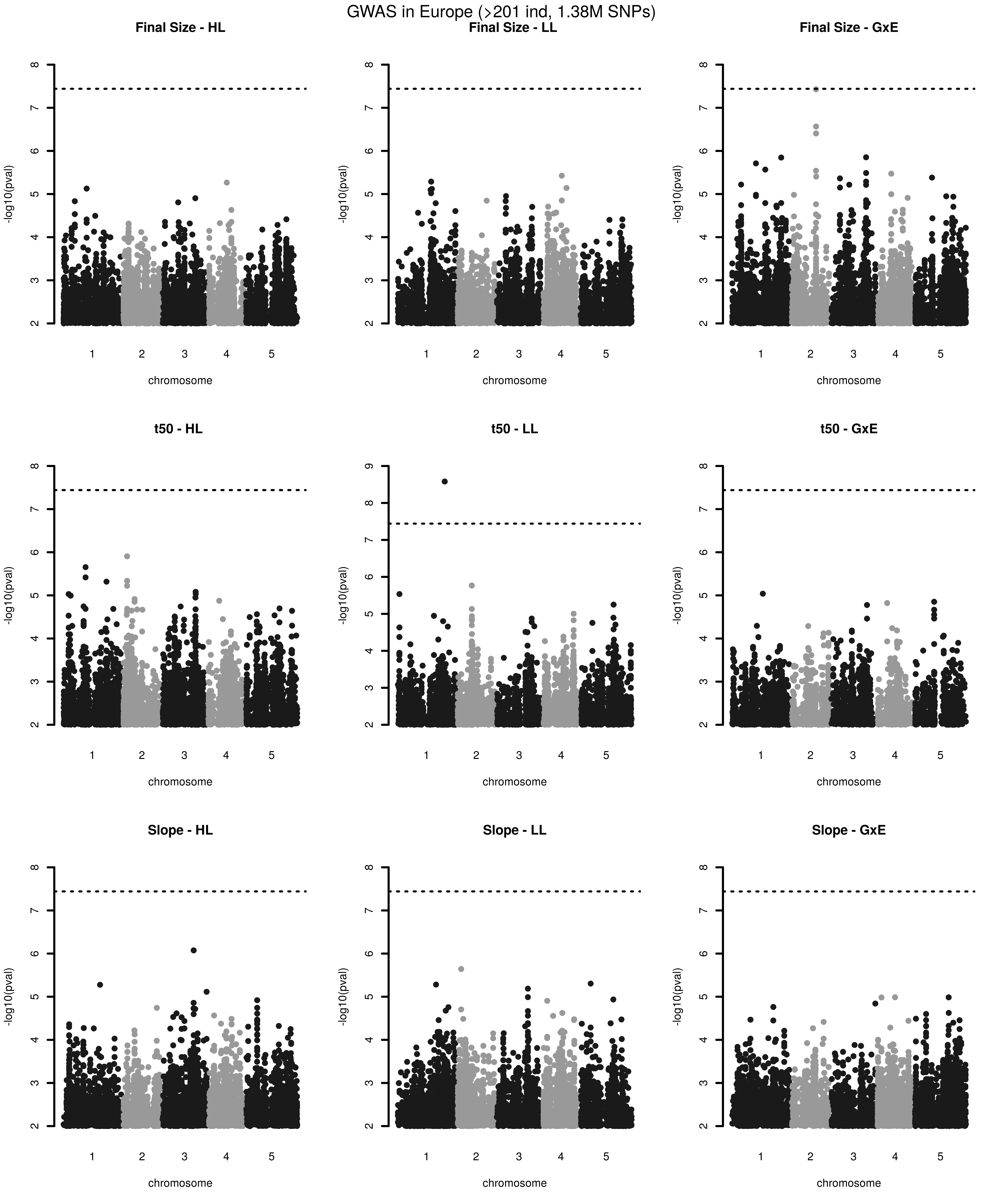

Supplement: S12 Fig — Manhattan plots using 201 (or more) genotypes from Europe (Spain and Northern Europe) as input. The traits are Final Size (upper row), t50 (2nd row) and Slope (lower row) in HL (left column), LL (middle column) and their GxE (right column). The dotted line denotes the 5% Bonferroni-corrected threshold. (TIF) [file pgen.1008748.s012.tif]

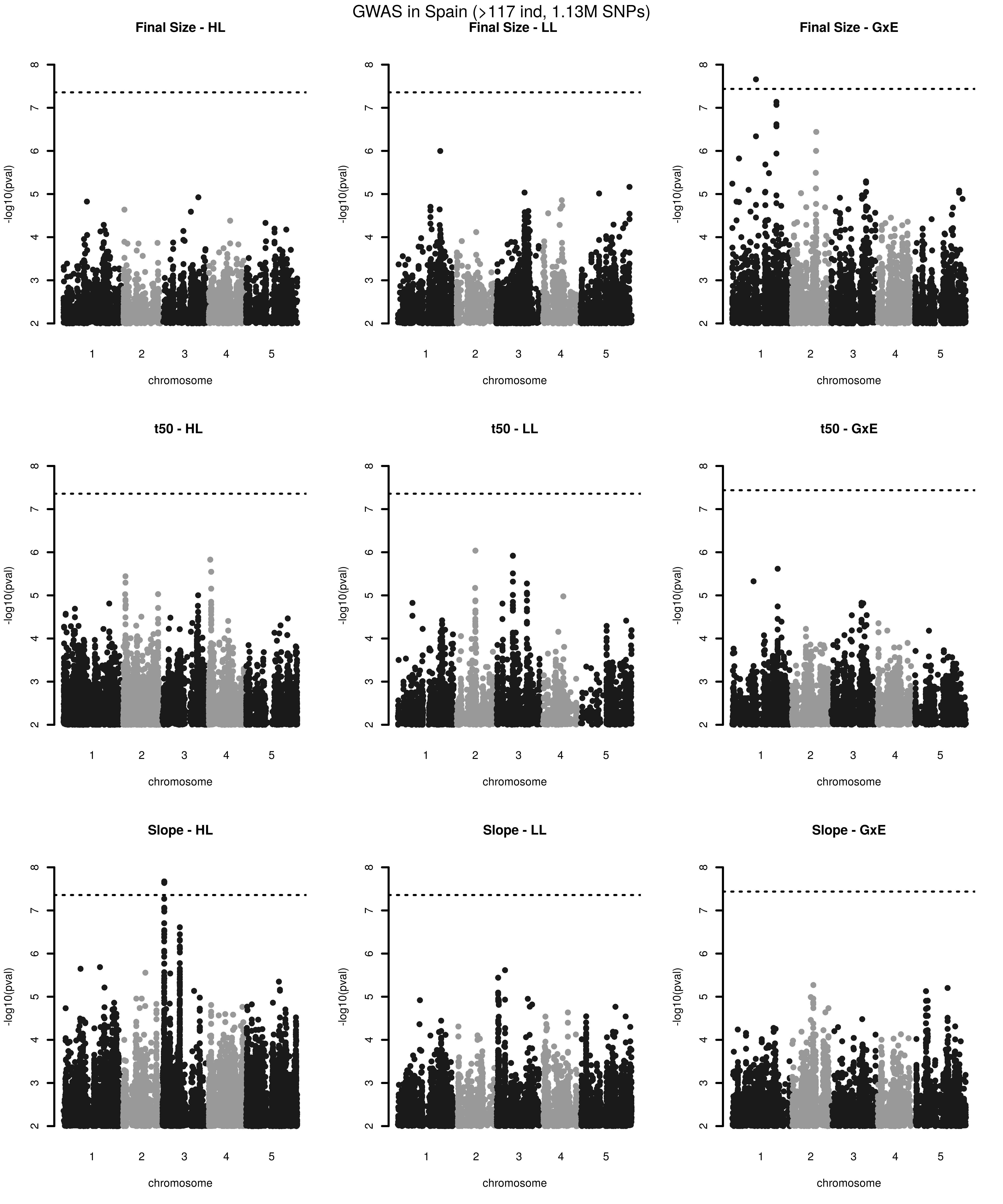

Supplement: S13 Fig — Manhattan plots using 117 (or more) genotypes from Spain as input. The traits are Final Size (upper row), t50 (2nd row) and Slope (lower row) in HL (left column), LL (middle column) and their GxE (right column). The dotted line denotes the 5% Bonferroni-corrected threshold. (TIF) [file pgen.1008748.s013.tif]

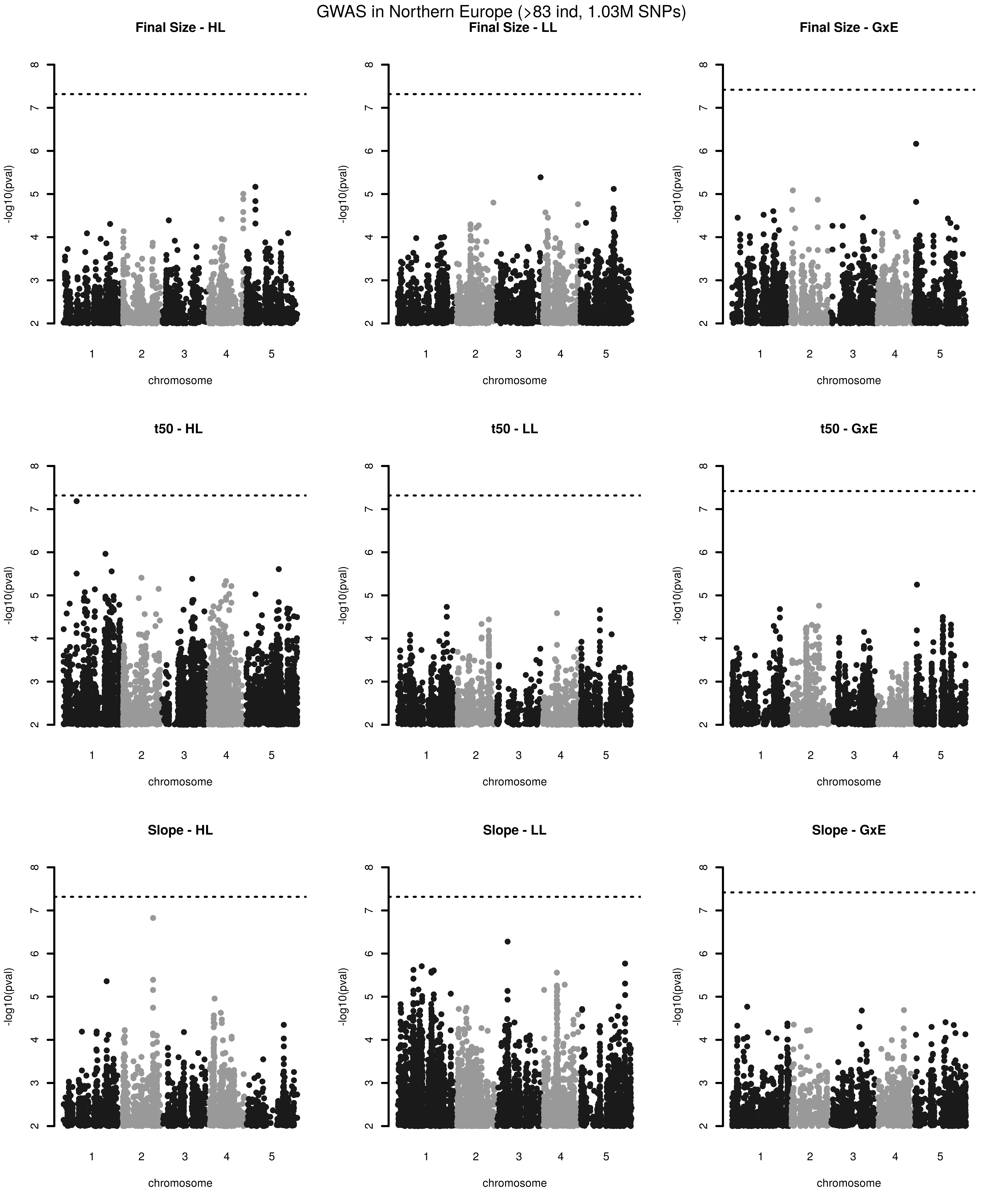

Supplement: S14 Fig — Manhattan plots using 83 (or more) genotypes from Northern Europe as input. The traits are Final Size (upper row), t50 (2nd row) and Slope (lower row) in HL (left column), LL (middle column) and their GxE (right column). The dotted line denotes the 5% Bonferroni-corrected threshold. (TIF) [file pgen.1008748.s014.tif]

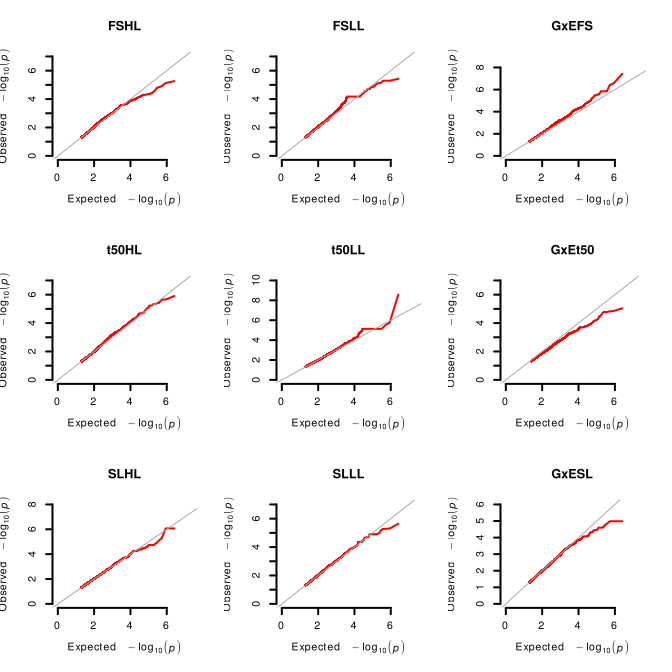

Supplement: S15 Fig — QQ-plots of GWAS using 201 genotypes from Europe (Spain and Northern Europe) as input. The traits are Final Size (upper row), t50 (2nd row) and Slope (lower row) in HL (left column), LL (middle column) and their GxE (right column). The grey line denotes the neutral expectation and the red line the observation from the data. The axes describe the expected (x) and observed (y) values for -log10(p). (TIF) [file pgen.1008748.s015.tif]

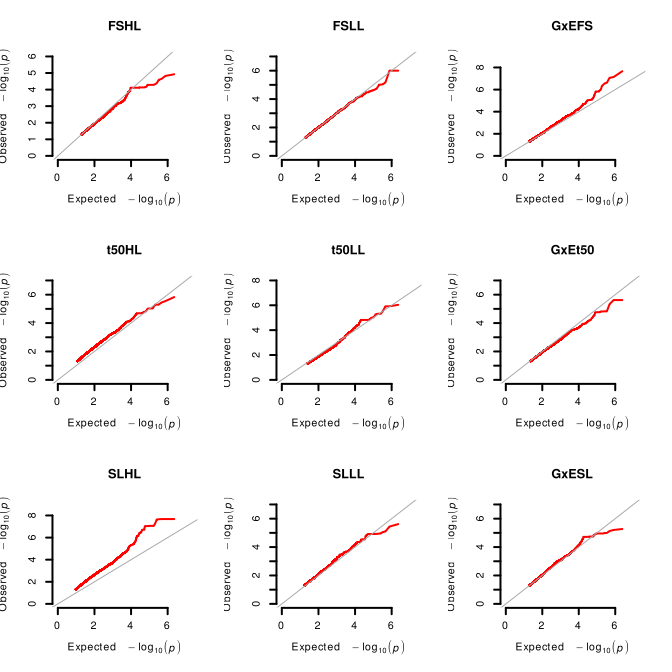

Supplement: S16 Fig — QQ-plots of GWAS using 117 genotypes from Spain as input. The traits are Final Size (upper row), t50 (2nd row) and Slope (lower row) in HL (left column), LL (middle column) and their GxE (right column). The grey line denotes the neutral expectation and the red line the observation from the data. The axes describe the expected (x) and observed (y) values for -log10(p). (TIF) [file pgen.1008748.s016.tif]

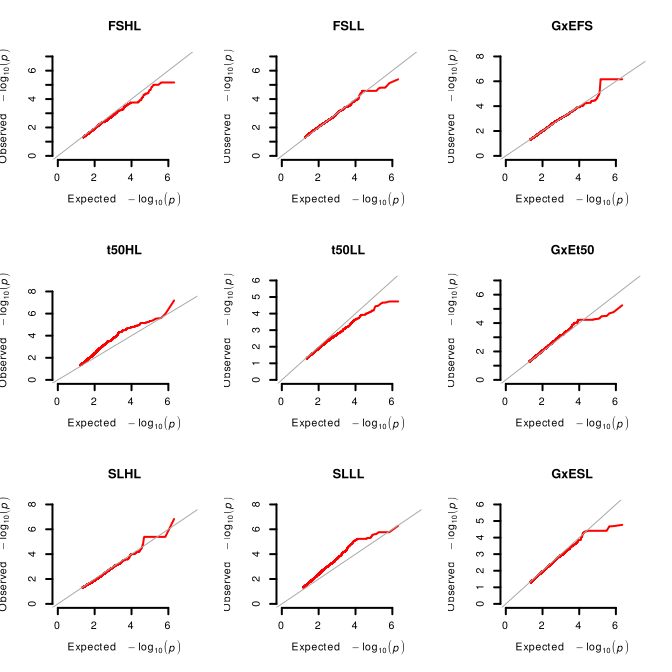

Supplement: S17 Fig — QQ-plots of GWAS using 83 genotypes from Northern Europe as input. The traits are Final Size (upper row), t50 (2nd row) and Slope (lower row) in HL (left column), LL (middle column) and their GxE (right column). The grey line denotes the neutral expectation and the red line the observation from the data. The axes describe the expected (x) and observed (y) values for -log10(p). (TIF) [file pgen.1008748.s017.tif]

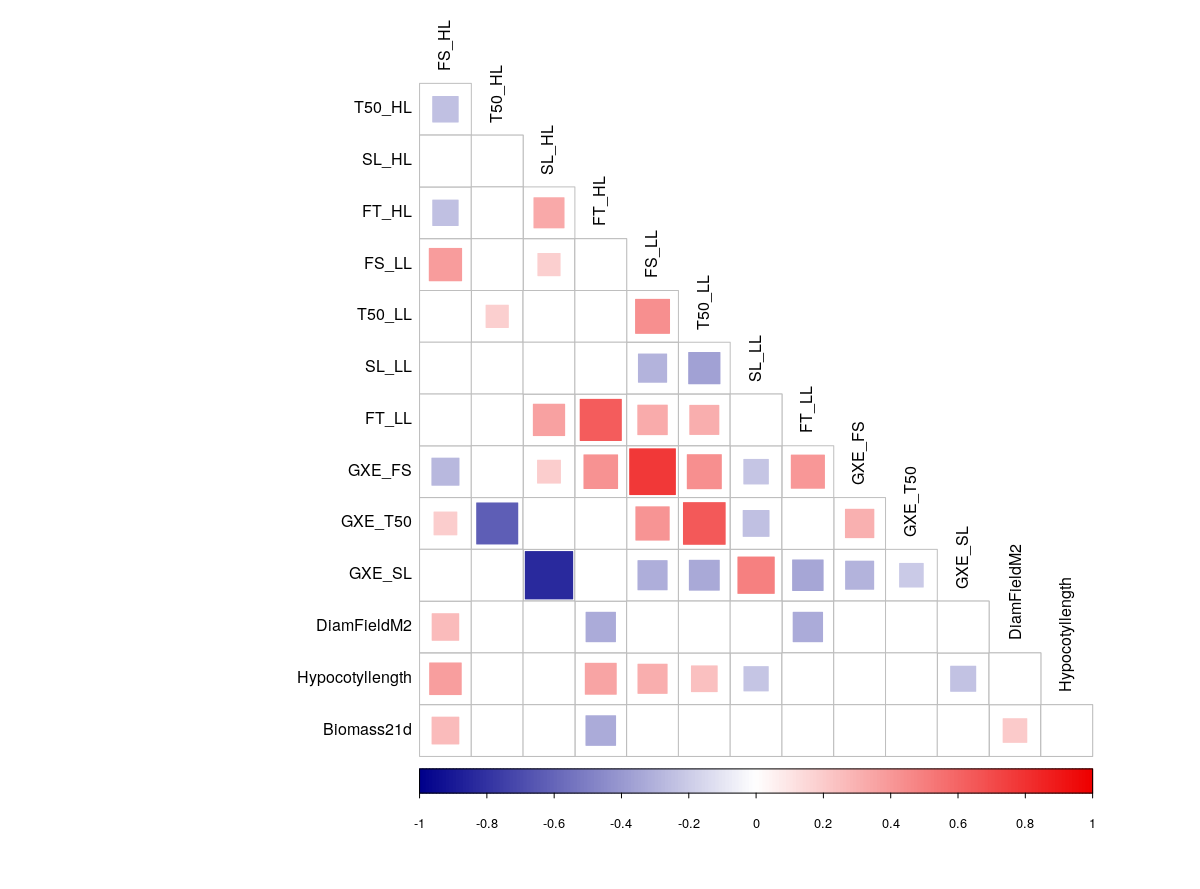

Supplement: S18 Fig — Pearson correlations for each pair of traits. Colored boxes show significant correlations (p<0.05 after multiple testing correction (FDR correction) and correction for populations structure (lmekin)) for 193 genotypes across experiments. The significance is illustrated by box size (larger box represents lower p-values) and the color shows the direction and strength of correlation. Abbreviations are: HL = high light, GxE = Genome x Environment interaction, LL = low light, SL = Slope, FT = Flowering time, FS = Final Size, DiamFieldM2 = Diameter in Field conditions after 2 Months, Biomass21d = Biomass in controlled (HL) conditions after 21 days. (TIF) [file pgen.1008748.s018.tif]

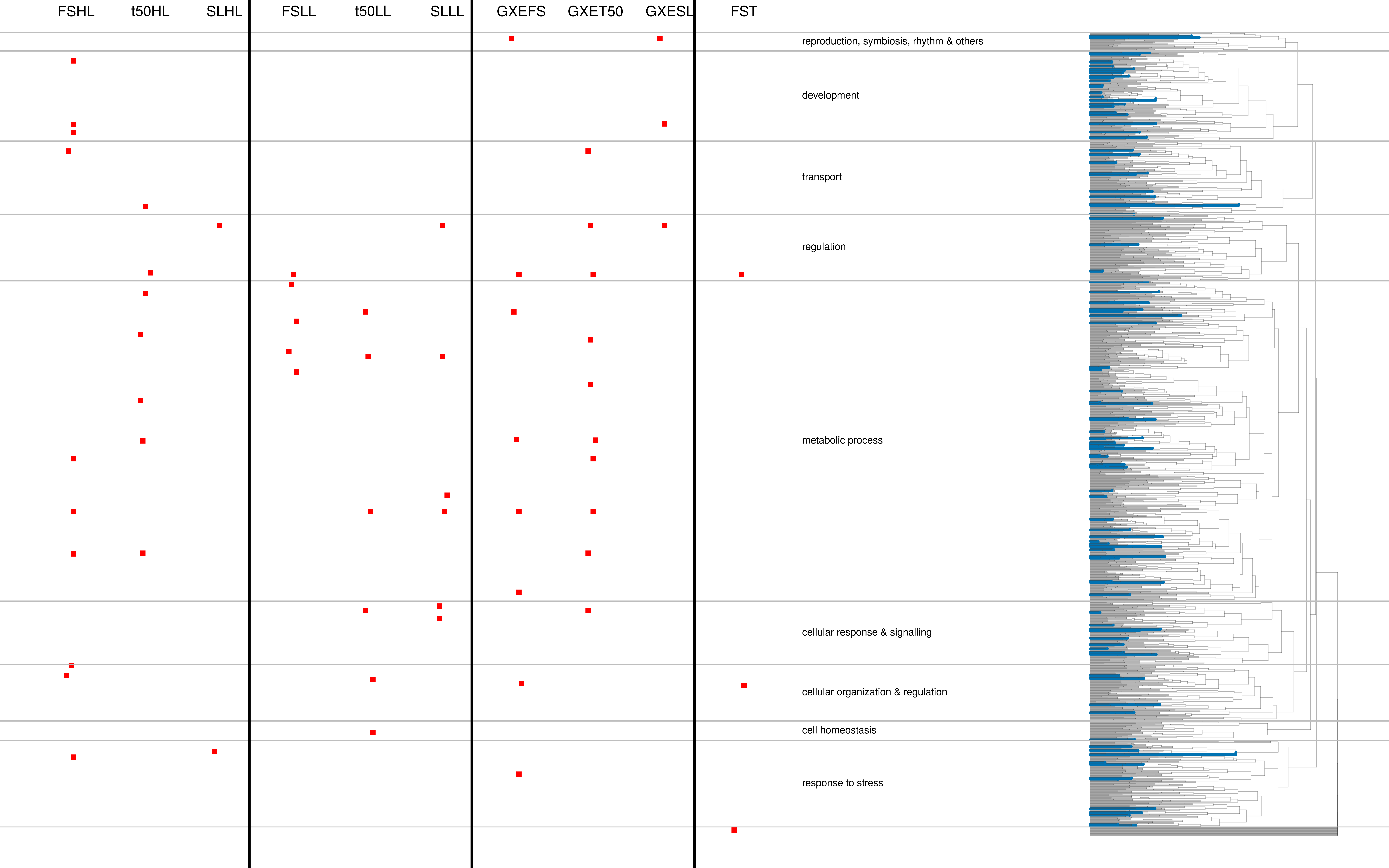

Supplement: S19 Fig — The enrichment is either based on ranking genes by p-value of the nearest SNP in GWAS (columns 1–9) or Fst of the gene (column 10). The GO terms are arranged into 9 clusters of similar function on the right side of the plot. Depicted are only enrichments with a p-value < 0.001. (TIF) [file pgen.1008748.s019.tif]

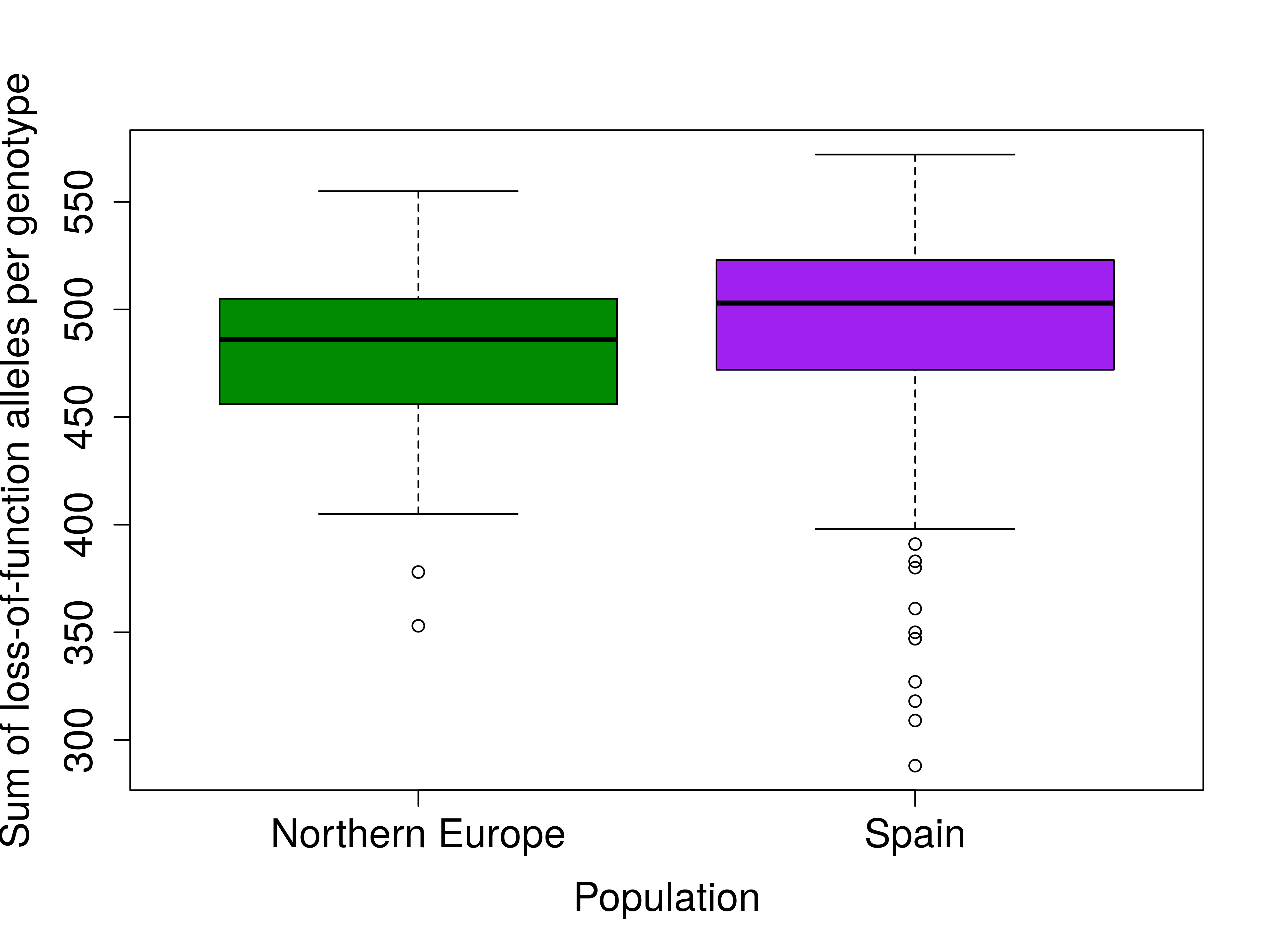

Supplement: S20 Fig — Based on data from Monroe et al. (2018). The sum of LOF alleles per genotype for Northern Europe (green, n =) and Spain (purple, n =). The regions were not different from each other (GLHT: z-value = 0.634, p-value = 0.526, negative binomial distribution). (TIF) [file pgen.1008748.s020.tif]

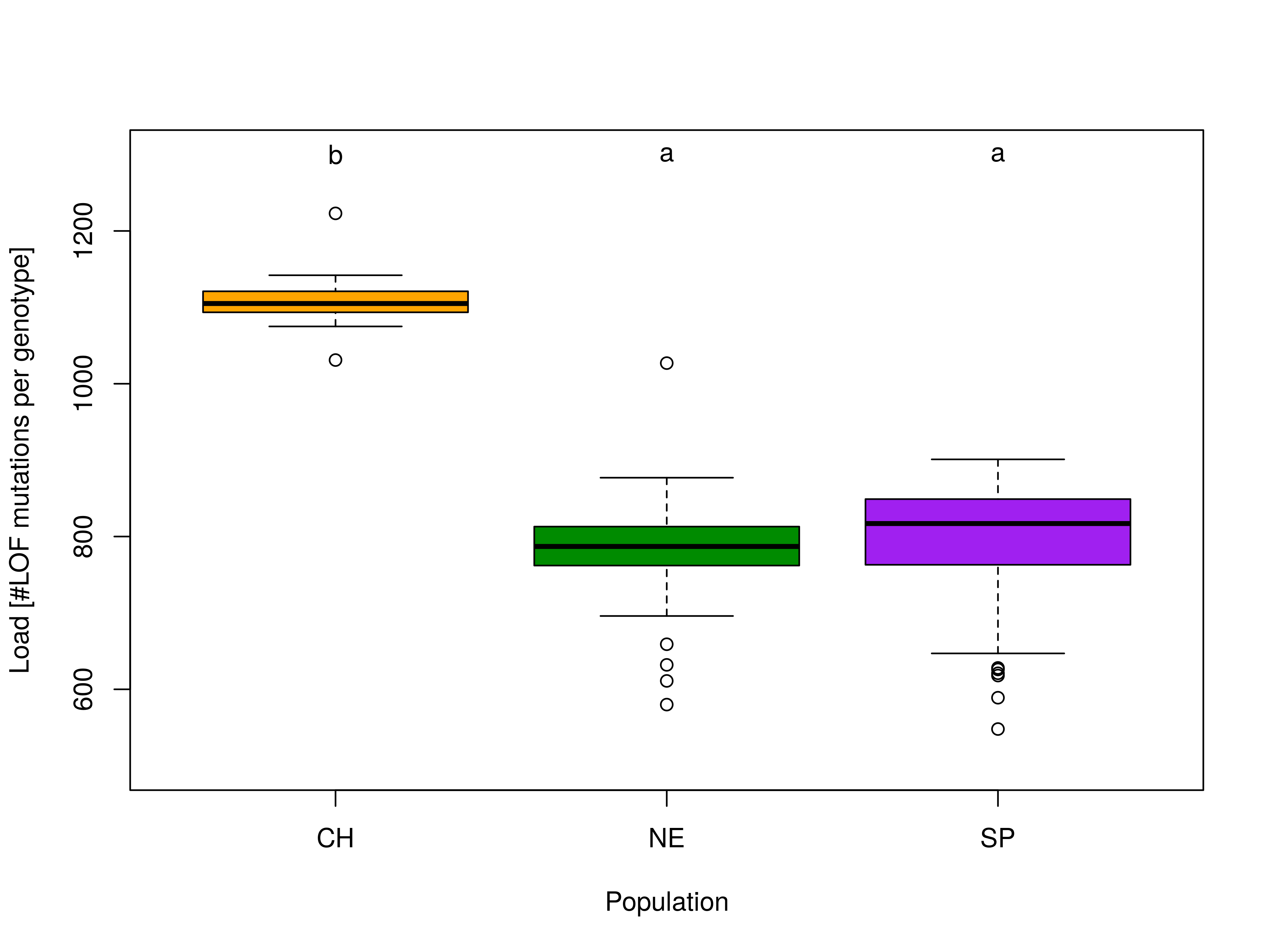

Supplement: S21 Fig — Based on data from Xu et al. (2019). Boxplot of the sum of LOF alleles per genotype for each region. Boxplots with different letters are significantly different according to Tukey’s HSD (p-value < 0.05). Region information: China (CH, n = 21), Northern Europe (NE, n = 84) & Spain (SP, n = 121). (TIF) [file pgen.1008748.s021.tif]

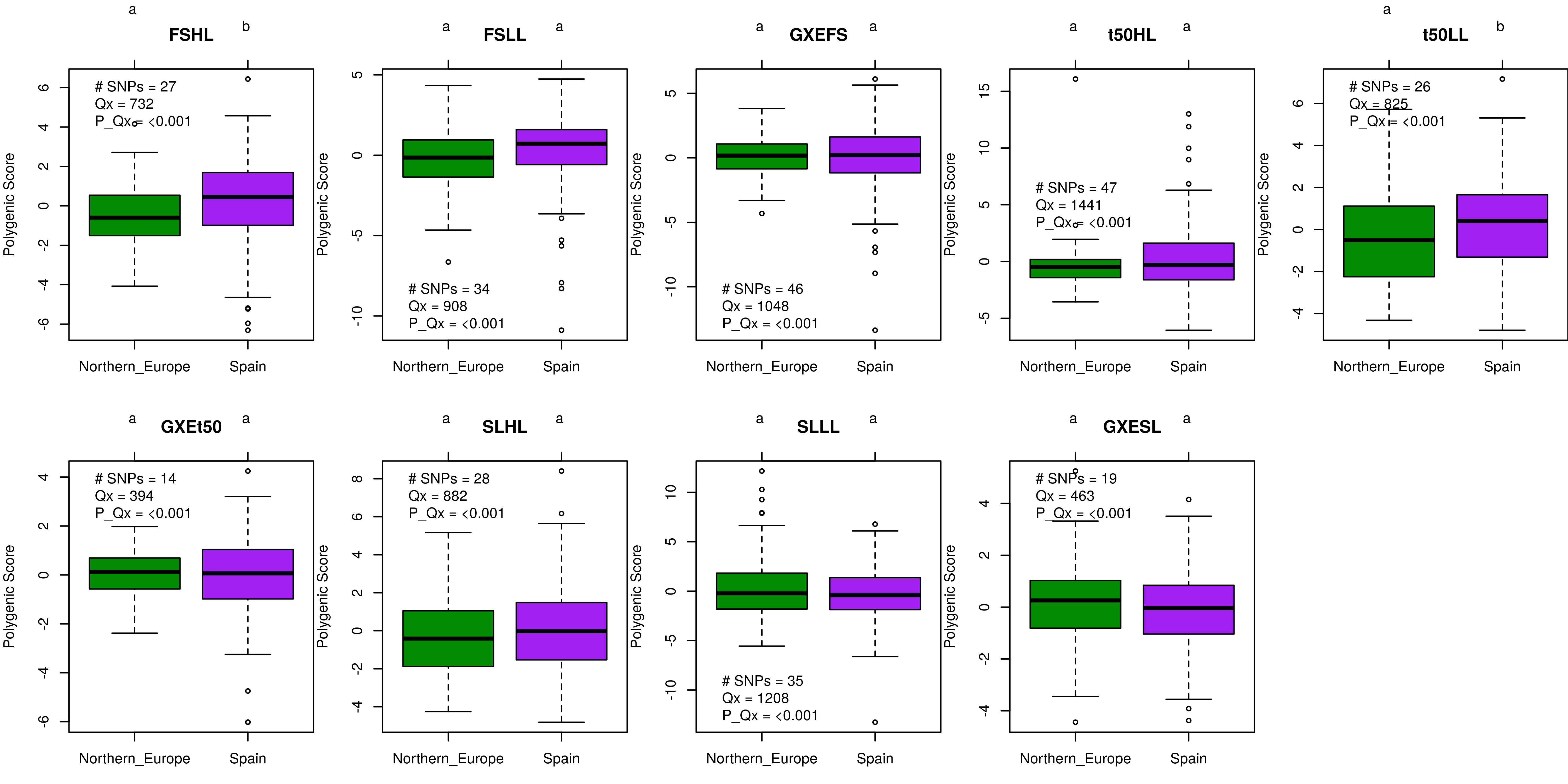

Supplement: S22 Fig — Summary results from the analysis after Berg and Coop (2014). Each boxplot depicts the polygenic scores of a trait for genotypes from Northern Europe (green) & Spain (purple). Boxplots with different letters are significantly different according to Tukey’s HSD (p-value < 0.05). Furthermore, the plot contain information about the number of SNPs used as input, the Qx score for excess variance in SNPs associated with the trait and the p-value of the Qx-analysis. Traits: FS = Final Size, t50, SL = Slope, HL = High Light treatment, LL = Low Light treatment. (TIF) [file pgen.1008748.s022.tif]

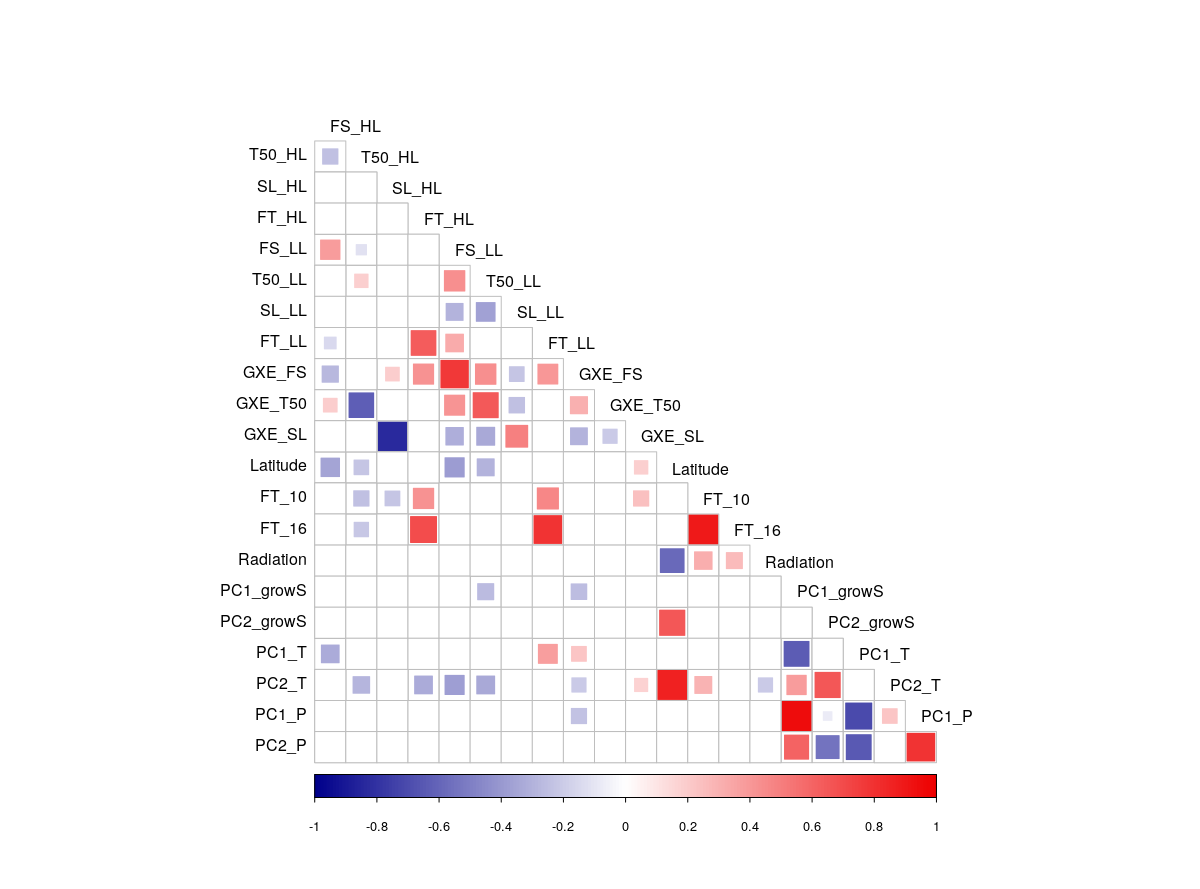

Supplement: S23 Fig — Pearson correlations for each pair of traits/climatic variable. Colored boxes show significant correlations (p<0.05 after multiple testing correction (FDR correction) and correction for populations structure (lmekin)) for 195 genotypes across experiments. The significance is illustrated by box size (larger box represents lower p-values) and the color shows the direction and strength of correlation. Abbreviations are: HL = high light, GxE = Genome x Environment interaction, LL = low light, SL = Slope, FT = Flowering time, FS = Final Size, DiamFieldM2 = Diameter in Field conditions after 2 Months, Biomass21d = Biomass in controlled (HL) conditions after 21 days, Radiation in kJ/m2/day, PC1/2_growS = Principle component 1 and 2 of all climatic data in the estimated growing Season (explaining 88.7 and 10.7% of the variance), PC1/2_T = Principle component 1 and 2 for climatic variables related to Temperature (explaining 98.1 and 1.3% of the variance), PC1/2_P = Principle component 1 and 2 for climatic variables related to Precipitation (explaining 89.8 and 8.22% of the variance). (TIF) [file pgen.1008748.s023.tif]
