# Supplementary material for: Polygenic adaptation of rosette growth in Arabidopsis thaliana
Source: PLoS Genet. 2021 Jan 25;17(1):e1008748. doi: 10.1371/journal.pgen.1008748 (PMC7861555; doi:10.1371/journal.pgen.1008748)
Supplement: Attachment — Submitted filename: PloSGenetics_Wieters_et_al_2020_response_to_reviewers_final.docx [file pgen.1008748.s036.docx]

**Feedback from section editor**:

Kirsten Bomblies
Section Editor: Evolution
PLOS Genetics


The reviewers clearly appreciated that this manuscript covers an interesting and important topic, and I agree and would like to see it get to the level that we can accept it. As is, however, there were substantial and I think largely very valid concerns raised. I was similarly puzzled in some sections. Of course this is complex data and a complex study, but I think the reviewers raise good points that will help hone and clarify the story.

We thank the editor for the opportunity to revise our manuscript. Altogether, we are very thankful for the detailed and high quality reviews we have received. We believe they have helped improve the clarity of the manuscript.

Questions regarding the statistical design, especially from Dr. Tavares (reviewer 3), made us realize that the rationale of the experimental design and its statistical analysis had to be explained much better. Our choice to use three growth parameters to describe the growth of each genotype was to facilitate the biological interpretation of differences between genotypes and/or environment. Indeed, these three parameters describe the maximum growth speed (slope), the duration of the growth period (t50) and the final size reached. We used linear models to calculate a genotypic value for each genotype in each environment following common practice in *A. thaliana* studies (see the AraGWAS database described in Togninalli et al. 2018). It is these values that we used as observations to understand the pattern of genetic variation and evolution of growth rate in *A. thaliana*. With the R script markdown we now include, we are confident that the reader will be able to understand all the details of our analysis.

We provide a detailed list of how we addressed each comment as well as a pdf version of the manuscript highlighting these corrections directly in the text. Note that, to answer the questions of the reviewers, we had to re-run several of our analyses. In doing so, we discovered a minor error in one of our dataset, which led to minor changes in pvalues in tables S5, S6,S9,S14 and S19. Note also that the title was slightly changed (we removed the word “variation”) and we added an Author’s Summary.

We hope that this revised version will convince the reviewers and the editor that our manuscript is suitable for publication in Plos Genetics.

Sincerely,

J. de Meaux

**Reviewer 1:**

Wieters et al., are using Arabidopsis thaliana rosette growth data from two different light

conditions to show that the genetic architecture of this trait is polygenic and that the variation

likely arose through adaptation.

The authors describe natural variation of growth by utilising a logistic function. The ecological

relevance of the growth chamber measurements is demonstrated with field experiments, which

is much appreciated.

Growth is a highly complex trait and it’s genetic architecture is, to no one’s surprise, polygenic.

Investigating whether that polygenic trait is adaptive is a much more complicated question.

Nevertheless, the authors managed to show that the variation in growth very likely arose

through adaptive processes rather than being the result of an accumulation of deleterious

mutations in certain populations.I appreciate the honesty of the authors to indicate that there still

are methodological challenges to consolidate their findings and that in their approach population

structure may still may act as a confounder.

This work is in my opinion an important example on how to study a polygenic trait in a common

garden setup. More specifically, the adaptiveness of growth variation is an important conclusion

for life history studies in Arabidopsis thaliana.

As always there are a number of unclarities in the manuscript which I would like the authors to

elucidate.

1) Can the authors clarify whether the correlation of FS with field plant diameter refers to

FS in high light or low light.

It actually refers to FS in HL. We clarified this in the manuscript on line 396.

2) Am I correct that the larger FS in LL is observed because rosette diameter is measured

and not rosette area? To my knowledge, shade avoidance is increasing petiole

elongation but not rosette area as such? This expectation should be clarified I think,

since it may mislead readers to think that plants show increased areas in LL.

In fact, leaf shape changes in order to achieve a larger rosette area and decrease leaf overlap. So we believe the reviewer is not correct, the shade avoidance reaction is a reaction to increase rosette area as such. In a test experiment preceding this work, we grew 17 genotypes and measured both rosette area and rosette diameter. The correlation was r=0.83 in high light (p=3.1e-05) and r=0.56 (p=0.01) in low light. Thus variation in rosette diameter is an indicator of variation in rosette area at both temperatures. We now briefly mention this result in the method section on line 197.

3) I miss an explanation why the genotypes were grouped in these exact four geographic

regions.

We have now clarified this aspect by rewriting the paragraph (line 147):

*“We chose 278 genotypes of Arabidopsis thaliana originating from 220 locations distributed throughout 4 regions for phenotypic analyses of growth rate variation (Northern Europe, Western Europe, Spain and Central-Eastern China, Table S1, Fig S1). A PCA confirmed that genotypes within these regions formed distinct genetic clusters (Fig. S2), whose specific evolutionary history has been previously documented* *[13,37–39].”*

4) To check whether plasticity or genetics is more important for growth variation, I think

genotype instead of region should be used in the MANOVA. This should not change the

results substantially but is more logic, to me at least.

Modified accordingly.

5) The section where growth and flowering time are being correlated is a bit confusing.

The authors show that FS is not linked to flowering time across the regions, using their

own flowering time data. Therefore the larger FS in Spain is not due to delayed

flowering.

Within regions there is no link with FS, but there is a region specific correlation with

slope in LL. It is not clear to me why the authors do not use their own flowering time data

here, but rather switch to public flowering time data (1001 genomes).

The region specificity of the correlation shows that rosette growth has a different

evolutionary history in different regions according to the authors. While it is plausible that

growth has different evolutionary histories in different regions, I don’t really see why this

correlation is a direct proof of this. Can the authors clarify the reasoning behind this

statement? There is an additional link between t50 and flowering time, only in Spain and only in HL. The authors conclude a trade-off between growth and flowering time here, but I miss the

relevance of this finding in the main conclusion of this section.

In my opinion, Fig3. could be simplified by focussing on the main messages/correlations

of this section. Additional correlations can be put in supplement. E.g. Vernalisation

requirement is not being discussed in the manuscript (I’m also not convinced that the

difference between flowering time at 10 and 16°C is a good proxy for vernalisation

requirement).

We agree with the reviewer that this paragraph is not directly relevant to the main message of this study. We therefore decided to simplify it to the one point we wish to make: Spain and Northern Europe differ in rosette growth average, but not in flowering time average. So the smaller rosette size observed in Northern Europe is not due to developmental differences that would stop growth earlier in this region.

We have 1) clarified paragraph on line 428, 2) removed figure 3 from the manuscript, 3) refer in the main text to two supplementary figures showing how the two regions that differ in rosette growth do not differ in flowering time.

6) While it is clear from the PCA in Fig. S3 that the Chinese accessions are genetically

distinct from the other accessions. I think the authors should still statistically validate

their hypothesis that the chinese accessions are phenotypically less divergent than

would be expected genetically.

To address this comment, we included a quantification of Fst difference between China and Europe.

Line 438:

“*The analysis of Chinese genotypes indicates that the phenotypic evolution of rosette growth does not scale with the extent of genetic divergence (Fst between Europe and China is 0.057 on average, with a standard deviation of 0.147 , and much greater than Fst between Spain and Northern Europe, KS test, D=0.39, p<2.2e-16). Since growth rate has not significantly changed despite extensive population divergence, it appears to have been constrained by stabilizing selection in the Chinese lineage.”*

7) ‘Local genetic variation in slope and t50, growth parameters which display moderate but

significant genetic variance, appear to be controlled by low-frequency variants of

comparatively larger effect, since some of them were associated above Bonferroni

threshold.’

Can the authors include allele frequencies and effect sizes to show this.

We apologize, we should have referred to table S7 (now S6), which provides allele frequencies and effect sizes. We have corrected this (line 461).

8) I have my doubts on the choice of training data and test data for the polygenic scores.

The polygenic scores were based on data from two replicates, for all genotypes. The

accuracy of the polygenic scores was then based on how well they predict the

measurements of the third replicate. I think this is only testing genetics vs plasticity. A

strong correlation between prediction and measurements of the third replicate means

that genetics, for which the selected SNPs are a proxy, are important in determining the

phenotype. The authors do show that the selected SNPs are doing better than randomly

selected SNPs. So there is definitely extra predictive value in the selected SNPs (which

is also to be expected since they were picked up with GWAS).

An alternative approach however, is to calculate polygenic scores based on a training

set of genotypes and see to what extent they are able to predict phenotypes of another

set of genotypes. This should remove the effect of linked genetic variation in estimating

the accuracy of the prediction.

We agree with the reviewer that it would be best if 80% of the data could predict the remaining 20%. Our dataset is however small, and shows an elevated degree of population structure. It therefore does not have the power to correctly predict unknown genotypes. Even in the case of FSHL the correlation of the 20% predicted phenotype to true phenotype was not significant (15 input SNPs, rho=0.07979094, p=0.6189). This is not surprising since genomic predictions are not efficient in the presence of population structure, even in much larger samples.

We have added one sentence clarifying that we tried using 80% of the data to determine effect sizes, but the obtained polygenic scores did not allow predicting the remaining 20% of the data.

Line 506:

*“Another and potentially more conclusive approach would be to use 80% of the data to identify SNPs associating with rosette growth and test whether they can be used to correctly predict the phenotype of the remaining 20% of the data. This approach usually does not perform well in structured populations* *[80], and indeed did not work here, even for the most heritable trait (not shown).”*

9) SNPs with p-value < 1e-4 were selected for polygenic score calculations. The functional

enrichment selected SNPs with p-values < 1e-3. Is there a reason to take different

significance thresholds for both analyses of the polygenic basis of growth variation?

The reviewer is right, this was not coherent. We now use the same threshold for functional enrichment and polygenic score calculations (p<1e-4). Results are unchanged.

10) It is unclear whether Qst/Fst comparisons are done only between Spain and

Northern-Europe or also between other regions. If Qst values were calculated for the

other regions as well then I don't think comparing to the Spain vs Northern-Europe Fst

value is valid.

They were done between Spain and Northern Europe. We clarified this in the main text (line 635) and in the legend of Table 2.

Minor comments:

- Check the reference numbering, I think they are in general off with one number.

We apologize for this problem caused by our reference manager when generating the last version of the manuscript. We unfortunately did not notice it. We have paid specific attention to the problem this time.

- The importance of the 10min far-red light pulse is not clear to me. Maybe add this to the

methods section.

This is to simulate sunset. We clarified this in the methods section (line 164).

- Method section says that slope was taken from the exponential phase, while the results

section talks about linear phase. It is from the linear phase. We clarified this in the text.

- FS, final rosette size and rosette diameter are sometimes used interchangeably, please

stick to one version. We replace final rosette size with FS. Rosette diameter is now used only when we talk about the rosette diameter measurement.

- Explain GLHT. General linear hypothesis testing, we clarified this in the methods (line 245).

- Supplemental data is already a bit figure-heavy, but I would still like to see the QQ-plots

for the different individual GWAS.

We added the QQ plots as a supplemental figure.

- Line 537. INIP1 should be NIP1. We corrected this.

- Line 553. Specify the sub-significance level. We clarified this.

**Reviewer 2:**

The manuscript from Wieters and co-authors sets out to understand the genetic basis of variation for rosette growth in the global population of Arabidopsis. To do so, the authors followed for several weeks the growth, approximated as rosette diameter, of a set of 278 accessions representing four broad geographic regions, grown in two light conditions (HL = high light, and LL = low light). They extracted three parameters from those measurements (FS = final rosette size; SL = slope of the exponential phase of the growth curve; and t50 = time at which rosette diameter is ½ FS) and used them to compare the different regional groups and run GWAS. Finally, the authors used a population genomics approach to look for signals of polygenic adaptation for growth in their dataset (Qx, Qst/Fst).

This manuscript addresses a very important topic in adaptation; the ability to match growth to environmental conditions and available resources is a quintessentially adaptive trait, especially for plants, but still very little is known about how variation for growth is genetically determined. The authors have collected a remarkable amount of phenotypic data, and have conducted an extensive set of analyses. I have, however, a few concerns with some of the analyses or their interpretation, and more in general on how the manuscript is framed. I am listing my main comments below following the organization of the manuscript, although that is not necessarily the order of importance.

1) Given the apparent focus of the paper on identifying difference in growth patterns (and local adaptation) between different Arabidopsis populations, I was a bit surprised by the author’s choice of light intensity to explore GXE interactions. While clearly light intensity has a major effect on rosette size, this seems a better setup to study variation in shade avoidance (as the GWAS results confirm) rather than to compare growth habits in different macro-climatic areas. The authors should motivate this choice (over, say, different temperatures, daylengths or water availability), and clarify whether the light intensities used are ecologically relevant (i.e. are consistent with differences between the regions examined in this study).

As the reviewer mentions, there are many environmental parameters that differentiate regions of the world. Most of these parameters (temperature, rainfall) are unpredictable. Others, like day-length, are completely predictable, but they reflect modes of environmental change. In both cases, identifying the correct conditions under which plants growth phenotypes can be assumed to reflect adaptive differences to relevant environments is challenging, especially since plants modify their life cycle to target the best season for growth. In contrast, light intensities differ systematically in Northern vs. Southern Europe, because of the angle of light incidence, which is always lower in Northern Europe. During the growth season, plants in Southern Europe are therefore constantly exposed to stronger radiation. We have clarified this in the introduction (line 138), and in the methods (line 165), and refer to figure S10 that shows regional differences in light radiation.

2) The authors use rosette diameter as proxy for growth. I understand of course that that was dictated by technical limitations; total biomass measurements are disruptive, and more comprehensive 3D shape measurements would likely be unfeasible at this scale. However, this means that they are exploring only a particular aspect of growth – depending on their flowering habit, Arabidopsis rosettes will continue to produce new leaves (and therefore “grow”) well past the point where they reach maximum diameter, since new leaves will overlap with older ones but not grow longer than them. Other ways of measuring growth would likely give different results, as it is hinted by the significant, but overall quite limited, correlation between FS and biomass or hypocotyl length. In particular, the effect of light intensity on growth would probably be quite smaller if aerial biomass was measured instead of rosette size; it seems likely that the increase of the latter under low light condition is largely due to the increased petiole length that is known to be associated with shade avoidance syndrome (see for example Sasidharan et al. Plant Phys. 2010).
This does not, of course, invalidate the results presented in the manuscript – however, the authors should make clearer what is the scope of their analyses early in the manuscript, and especially in the abstract.

We have made clear in the abstract and in the results that plant diameter was used as a proxy for monitoring plant growth over time (line 58, line 345). We also followed the recommendation of the reviewer and mention that the shade avoidance response may explain why plasticity is the major source of phenotypic variation in our dataset (line 407).

3) The authors use a polygenic score analysis and GO enrichment analysis to show that, while only two SNPs pass the very conservative Bonferroni-corrected significance threshold, the GWAS results describe nonetheless the polygenic basis of variation for the examined growth parameters. I am particularly skeptical of GO enrichment analyses; unless they show clear cut patterns, they are labile to different interpretations. The authors interpretation of the GO enrichment results is that “All traits showed functional enrichment within gene ontology (GO) categories related to growth, confirming that these genetic associations were biologically relevant”. However, many of the enriched categories seem unlikely to be directly associated with variation for growth (e.g. “pollen exine formation”, “nuclear chromosome segregation”, “protein modification by small protein removal”, to name a few). A notable exception is GXE FS, for which the four most significantly enriched categories include “shade avoidance” and other three categories which can be plausibly linked to aerial growth/rosette size. Given how noisy the results are, I would suggest the authors remove this analysis, or limit its discussion to GXE FS.
These considerations are also valid for the comparison between genes with elevated Fst and SNP associations. While this analysis uses a different approach, I would have still expected similar results to the previous analysis for SNP associations; however, there is almost no overlap in

enriched GO categories between the two analyses (or, for that matter, between the same parameter measured in different light conditions), which appears to further undermine their value.

We agree with the reviewer that GO enrichment categories are sometimes not interpretable, and one is left with no other option than to cherry pick what makes immediate sense (such as shade avoidance), leaving out what is harder to interpret. Some strange GO enrichment may be false signals, but they may well be real signals with wrong names. Indeed, category definitions are inherently biased by the experimental data available, and there is no such category as “rosette growth”. Unfortunately, at present there is no alternative for evaluating the functional signal given by GWAS. In this context, our strategy has been to determine an adhoc pvalue threshold that is below the pvalue that could be obtained based on random data (line 334). We had picked the p-value threshold of 0.008 because it is in 99.999% of the cases below the lowest p-value you can obtain for GO enrichments run for 1000 random SNP sets of equal size. We now explain this in detail this conservative threshold in the methods (line 333). We also toned down our reference to the functional enrichments in the abstract (line 68).

4) As for the polygenic scores, I readily admit to not having extensive experience on the topic, so I might be missing or misunderstanding something – in which case I apologize beforehand. It seems to me, however, that using polygenic scores from two replicates to predict the phenotype of the third replicate is functionally identical to predicting the input data – with the accuracy of the prediction being dependent on how consistent between replicates (i.e. heritable) a trait is. Minimally, the authors should provide evidence that the predictions are significantly different from what you would get for random SNPs (possibly generating a null distribution for each parameter), and clarify the interpretation of the results. While the authors do provide one example using random SNPs for FSHL (Table S11), it is not clear how these SNPs have been selected, and it is not a direct comparison (it appears that the polygenic scores for the random SNPs have been calculated using all three replicates, and not to predict the third replicate as is the case for the results in Table S8).
An even more convincing experiment would be to remove a few accessions from the dataset, perform GWAS and calculate polygenic scores on the rest of the accessions, and then use those polygenic scores to predict the phenotype of the remaining accessions. Given the relatively small sample size, such analysis might however be under-powered.

We totally agree with the reviewer and for this reason we had tried to be very careful in our interpretation. In fact, genomic predictions based on polygenic scores still perform very poorly in structured samples, even in humans. We have added one sentence clarifying that we tried using 80% of the data to determine effect sizes, but the obtained polygenic scores did not predict the remaining 20% of the data (line 506).

5) These observations do not mean that sub-significant association are not biologically relevant; nevertheless, unless the authors can provide a more convincing proof, they should acknowledge the possibility that, especially for the low heritability parameters SL and t50 (0.07-0.02, Table S4), GWAS results are extremely noisy and might not be informative. This, however, could have significant consequences on the interpretation of further analyses that rely on GWAS results (Qx). The authors could consider to only focus on the more heritable FS parameters for most of the analyses.

Heritabilities were low, but not as low as the reviewer writes.. Pseudoheritabilities can be much lower than trait heritabilities, if genetic variation in a low heritability trait is controlled by a few variants (which is the case for t50). In addition, growth rate being such a complex trait, it is to be expected that not all parameters that describe it have the same heritability. Initially, we did think about focusing the manuscript on final size, but t50 reveals additional aspects of plant growth variation that should not be ignored. For example, the association between t50 and LOF allele variation revealed one of the strongest association in the study. This is not the case for slope, but slope did not have a much lower heritability than t50. In addition, the Qst analysis (which is not dependent on the genetic architecture of the trait) indicates a weak signature of local adaptation for FS, but also for t50. For these reasons, we opted for analyzing all three parameters irrespective of their heritability. We hope that the reviewer will agree with us that we have made sure to remain cautious in our conclusions.

Other points:

6) The authors should give more information on how the accessions were chosen, and how the different European regions were defined - was it based on previous genetic characterization (although West European accessions seem to cluster with either Spain or North European ones in the PCA in Fig. S3), or on climatic differences? The Western European group of accession in particular seems not clearly defined; in Fig. S1, one German accession is classified as West European, but all German accessions are classified as North European in Table S3. At different points of the manuscript is not always clear which regional groups are included in which analyses. Since Western European and Chinese accessions are not included in most analyses after the GWAS, it might be helpful to specify at that point that the remaining analyses will focus on the Spain-Northern Europe comparison, and specifically mention the other groups whenever they are included.

We modified the text according to the suggestion of the reviewer. See also answer to reviewer 1 above. Note that there was a typo in Table S3, which led to a discrepancy with Fig. S1.

7) Data for biomass, hypocotyl length and FS in field experiments are used in comparisons with the rosette growth parameters used throughout the manuscript, but they could be integrated further in the analyses (GWAS), since they would provide additional information on growth variation. Either way, since they are used in the manuscript, data for those experiments should be added to the supplementary material (I don’t think they currently are).

The data on these phenotypes is in Suppl. Table 3. We however prefer to focus the manuscript on the GWAS analysis of our main experiment. These other datasets are currently included in another manuscript investigating other aspects of phenotypic variation under natural conditions.

8) I am not sure that using the flowering time data from the 2016 1001 Genome Consortium paper to test correlation between flowering time and the growth parameters described in this paper is appropriate here – those plants were not only grown at different temperatures, but also with a different light cycle (16 h of light and 8 of dark vs the 12 h of light, 20ºC, and 12 h of dark, 18 ºC, used for this manuscript). While the general trends would be similar, as the relatively strong correlation between the different sets of flowering time data shows (Figure S19), the differences could be large enough to confound the analysis. While FT16 and FT10 are the next best thing, since flowering time was not measured for all accessions in this study, these limitations should be acknowledged.

As mentioned above, we have considerably modified this paragraph and focused it on what is essential for this manuscript, namely that the differences in plant growth between Spain and Northern Europe are not reflecting differences in flowering time.

9) Related to the previous point, the description and presentation of Figure 3 should be improved. Are the thickness and length of the lines proportional to the significance and/or strength of the correlation? If so, the information should be included in the figure (even if, as the figure legend mentions, numeric values are reported in Table S6). The striping is also difficult to see on the red lines.
It also not clear why the “VER” parameter is included, since it is not mentioned elsewhere and it does not seem particularly germane to the analyses in the manuscript. If it is kept in the figure, it should be addressed in the manuscript, and a reference supporting the correlation between FT16-FT10 and vernalization requirements should be added (I could not find mention of that in the 2016 1001 Genomes Consortium paper, but it is a very expansive paper).

We thank the reviewer. Indeed, we forgot to explain how “VER” was calculated. It is the difference in flowering time between FT16 and FT10. Accessions whose flowering time was delayed at 16°C compared to 10°C are accessions that tend to need vernalization (VER>0), whereas those that flower faster (VER<0) don’t. However, as mentioned above, we have removed this figure from the manuscript.

10) While I have little doubt that growth rate is constrained in Arabidopsis, I am not sure selection is necessarily the only explanation for the observation that growth rates in Chinese populations are within the range of variation within European populations – there could be physiological constraints, or European populations, not having experienced (as a whole) the same bottleneck as Chinese population, could be genetically more diverse (which I believe is the case) and cover a broader range of the phenotypic space for growth. The authors should provide a more detailed explanation of why they think selection plays a major role in this pattern.

We thank the reviewer for this comment. It helped us improve the corresponding paragraph (line 432). Note that our reasoning is based on average genetic differences *between* regions, not on the level of genetic variation within region. The genetic divergence between regions does not scale with the phenotypic divergence between these regions. This is not related to the amount of variation you have within regions.

We now write (line 433):

*“Despite a long history of population isolation that was magnified by a strong bottleneck after the last glacial period* *[38,71], the growth rate of Chinese genotypes was comparable to that shown by most European genotypes (Table S5, Fig. 2 a-d). Under LL conditions, Chinese genotypes showed lower t50 and FS values only when compared to Spanish genotypes (Table S5, Fig. 2 a-d). Under HL, genotypes from China did not differ significantly from those from any other region (Fig. 2, Fig. S11). The analysis of Chinese genotypes indicates that the phenotypic evolution of rosette growth does not scale with the extent of genetic divergence (Fst between Europe and China is 0.057 on average, with a standard deviation of 0.147, and much greater than Fst between Spain and Northern Europe, KS test, D=0.39, p<2.2e-16). Since growth rate has not significantly changed despite extensive population divergence, it appears to have been constrained by stabilizing selection in the Chinese lineage. “*

11) It would be helpful to compare the GWAS results for GXE FS to those for the shade avoidance GWAS (measured as hypocotyl elongation) from Filiault and Maloof, PLoS Genetics 2012.

This is a good suggestion, however, the comparison is difficult. It is not the same set of genotypes and GWAS studies can be very sensitive to the genotype sample. Unsurprisingly, the main candidates were not retrieved (when population structure was accounted for), and both the phenotype measured and the growth conditions were different. However, we agree with the reviewer it is important to cite it, since it also studies reports a polygenic architecture. We now mention it on line 529.

12) I found the LOF GWAS to be an interesting approach, especially since it greatly simplifies candidate gene validation – one could quite safely assume that the LOF, and not some more complex regulatory change, is causal for the phenotypic differences. While I do not expect the authors to start a whole experiment just to humor me, it would be neat and relatively straightforward to knock out NIP1 in a few accessions carrying a functional copy (or, even easier, getting a T-DNA mutant line, since I am guessing the Col-0 allele is functional) and check if t50 is indeed affected.

The reviewer is right, but there is no T-DNA mutant line available for this gene. We are investigating phenotypic change in knock-outs of a gene that is direclty interacting with NIP1, but this study is still in its infancy.

Minor points:

- Line 49-52: This sentence is a bit confusing, the same concept is expressed more clearly elsewhere in the manuscript.

We modified the sentence. It now reads:

*“For both light regimes, phenotypic divergence between the most distantly related population (China) and the various regions in Europe is smaller than the variation observed within Europe, indicating that some level of stabilizing selection constrains the evolution of growth rate.”*

- Line 224-226: numbers sum up to 231, not 235.

Thank you for pointing this out, we corrected it.

- Line 386-397: Figure S7 only shows that flowering time (as FT16 from the 2016 1001 Genome Consortium paper) is not significantly different between North European and Spanish accessions. Directly showing correlations between FT16/FT10 and the parameters analyzed in the manuscript would be more informative (this regardless of my previous comments on whether using those data is appropriate).

This comment is no longer relevant since we have considerably streamlined the discussion of the relationship between flowering time and rosette growth variation.

- Line 460: should be 22-37 (in Table S8, there are 37 SNPs in the analysis for t50LL).
Thank you for pointing this out, we corrected it.
- Line 537: should be NIP1, not INIP1.
We corrected this.
- Line 553: should be 14-47 (in Table S11, there are 14 SNPs in the analysis for GXEt50).
Corrected.
- Line 563-565: this sentence is likely mis-placed (it is repeated almost verbatim immediately below).
We modified this sentence.
- Line 611-612: While this sentence is technically correct (three Western European accessions were included in the GWAS, and Chinese accessions were included in the LOF analyses), most (population) genetic analyses focus almost exclusively on the Spain/Northern Europe comparison.

We modified the sentence in the conclusion and now only refer to three regions (China, Northern Europe and Spain). (Line 658)

- Line 614-615: this should probably be re-phrased to specify that light intensity has the strongest effect among the factor tested in this study (other environmental factors not tested here might have even stronger effects).
Modified accordingly.
- Lines 626-628 and lines 629-630: the two sentences are almost identical.
Thank you for pointing out this typo.
- Figure 6. “Horizontal lines” should be “vertical lines”.

We corrected this.

**Reviewer 3 (Hugo Tavares)**

The manuscript submitted by Wieters et al. describes the variation of plasticity in rosette growth in Arabidopsis in response to light conditions. By taking weekly photographs of the rosettes of individuals growing under controlled growth chamber conditions, the authors were able to track the growth dynamics of individual plants over time. Using a collection of Arabidopsis accessions originating from a wide range of locations, the authors assessed the genetic variation in growth parameters extracted from their data. This geographically diverse set of accessions was used to explore how differences in growth may have been shaped by ecological and/or evolutionary processes, which seemed to be the main aim of this work. From the growth curves of each individual, the authors extracted metrics such as growth rates, final sizes and the time it takes to reach certain phases of growth. By running several GWAS scans on these metrics, the authors largely failed to identify significant associations, despite a reasonable heritability for some of them. The authors conclude this is likely due to the polygenic nature of these traits and explore this issue by fitting multi-locus models to obtain polygenic scores. They also explore correlations between sub-samples of accessions (based on geographic region) and the prevalence of loss-of-function alleles, as well as correlations with patterns of differentiation to discuss their results in light of evolutionary processes such as range expansion and local adaptation. Collecting such time-resolved growth dynamics for a wide range of accessions seems like an important contribution to understanding this complex and integrative trait, which is modulated both by external (environmental) and internal (developmental) signals. Not only is this trait important as a fitness component of plants, but also of major interest for example in crop research. Therefore, understanding the underlying genetics and plasticity of such a trait is of relevance for plant geneticists specifically, but also for those interested in the quantitative genetics of complex traits more broadly, and the dataset produced by the authors seems like a very valuable contribution to the field.

We thank the reviewer for this accurate summary. We were happy to hear this positive opinion.

The authors did a good job at finding ways to look at their data from multiple angles. However, I have some major conceptual and analytical concerns about the work, which made it hard for me to assess whether many of the authors conclusions followed from their data. I also feel like the manuscript could be expanded, in particular by having separate results and discussion sections. I will first give a broader overview of my criticisms, followed by detailed suggestions, which I hope help to improve the analysis and interpretability of these data.

**Major points**

1) Conceptually, I felt that much of the authors focus and attention was misplaced. Too many of the results seemed to be forced into answering evolutionary/ecological issues. I was not convinced by the argument that the traits measured under growth chamber conditions necessarily reflect what happens in the field. Personally, I think the data collected is more suited to address fundamental quantitative genetic questions of how to dissect the genetics of complex traits (which the authors do, but choose not to focus their narrative on as much). I do think ecological/evolutionary questions are important, but these should perhaps be presented as more speculative in a separate discussion section.

The reviewer is right: our main interest is in the evolution of growth rate and its importance for ecological adaptation. The sample of genotypes and the analyses we conducted were therefore guided by this question. Using GWAS to study and predict adaptation is a popular approach currently, but too few studies discuss its pitfalls in the way we do. We therefore do not agree that the focus of the paper is misplaced. Our data can certainly also be used for dissecting the genetics of complex traits, but we point to the fact that other groups are better positioned for tackling this question (see e.g. Marchadier et al. Plos Genetics 2019).

2) I also felt that the authors need to better clarify and the present data in a way that distinguishes between genetic and non-genetic plasticity components. Since they are both plasticity in a sense, but at looked at from different levels: at the level of light treatment (expressed as the average response of genotypes to light), or as the level of individual genotypes (as the variance in responses between accessions). There is largely no discussion of these gene-by-environment issues, and how they may or may not explain the lack of association hits in GWAS.

We were not sure about what the reviewer criticized here. We used two light conditions to reveal growth plasticity but also tested for the presence of genetic variation for plasticity (GxE see Table 1). May be the fact that we now show the significance of GxE in table 1 will address the criticism that the reviewer tried to voice?

3) I also found the discretisaton of the data into “regions” possibly problematic. I can see the rationale for this based on the geographic (Fig S1) and genetic (Fig S3) distances. However, there is a large continuum in these groups, with some individuals labbeled as the same “region” being farther apart than individuals across regions (both in geographic and PCA space). The exception is perhaps the Chinese accessions, which seem diverged enough to justify such partitoning. I am not saying that there are no differences between accessions of different geographic origin, I’m just concerned that slicing and dicing the data in relatively arbitrary ways may cause spurious differences to be picked on and over-interpreted (or, for that matter, failing to find differences, where they might exist but be masked in this grouping of the data).

The reviewer questions whether it is reasonable to consider the regions we have defined as relatively separate evolutionary units. These units are supported both by their geography and by their genetic set up (PCA). In addition, separating these units does not imply that there is no shared variation. The Fst distribution quantifies the degree to which they have diverged. Finally, population genetics analyses have shown that Northern and Southern European regions form metapopulations with distinct dynamics (see e.g. Kronholm et al. 2010 or Fulgione et al. 2018), not to mention chinese *A. thaliana* population that are deeply separated from European populations.

In order to clarify this aspect, we have partially rewritten the description of the material used (line 147).

4) Finally, at a technical analytical level, the authors’ approach to analyse their growth data left me unsure about the uncertainty associated with the rest of their analysis**.** If I understood it correctly, a logistic curve was fit to each individual separately. Then the parameters of this fit were again fit to a linear model to account for blocking effects. Then, a third model was fit to the mean prediction of the previous model, to get GxE components. And finally, the authors then use Tukey tests to evaluate differences in the last model’s parameters across groups. Although I can see the rationale used, the problem is that each of these models estimate a parameter with some error. However, using the authors’ approach this error does not propagate from one model to the next (because the authors only use the mean prediction of the previous model as input to the next model). Ideally data modelling should be done within a single statistical model, so that the error correctly propagates to all levels (block, accession, treatment). I make some extensive suggestions for possible analysis below.

The comments of the reviewer were very useful to realize that we had to 1) describe the experimental set up better and 2) describe our statistical pipeline with more accuracy.

Our experimental had to be set up in two separate trials, one in HL the other one in LL, each with 1 individual of each genotype per block and 3 blocks. This is because it is not possible to grow a plant under HL next to a plant growing under LL.

Our statistical analysis has been designed to facilitate both the biological interpretation of the data and its comparability to existing datasets in *Arabidopsis thaliana* (AraGWAS, Togninalli et al. 2018). Because our experimental design is fully nested, we can perform each of these steps separately. We fit a growth curve for each replicate of each genotype, and then extract means for each genotype, after accounting for block effects. It is these genotypic means that are used as measures of phenotypic variation in the AraGWAS database. We thus provided genotypic mean within each of the two environments. We then also analyzed genotypes simultaneously in both environments in order to extract estimates of genetic variation in growth plasticity. It is these genotypic values that form the independent observation characterizing the 4 metapopulations we compared.

We applied the model proposed by the reviewer, but it did not allow fitting the data better than our model. It is possible that more expert prior tweaks could have overcome the problem, but we felt that the linear models we used are much more straightforward, especially since GWAS rely on estimates of the fixed genotype effects, but do not consider the variance around the estimate.

To clarify how our phenotypic analysis was conducted, we now 1) restructured the paragraph describing statistical analyses, so that the extraction of growth parameter and the calculation of heritabilities are now part of the same chapter. We explain therein that our experiment is fully nested (line 154), so that data can be processed in successive steps and 2) provide an annotated R markdown of the analysis we have conducted. We give more details on these changes below.

**Introduction**

L68: Variation may also simply be a consequence of random drift, or occur due to a correlation with other traits themselves under selection. Also, plasticity itself may be selected for (under certain circumstances), so the apparent opposition between “plasticity” and “adaptation” in this paragraph could be misleading.

The reviewer is right. Obviously drift also plays a role and this is why adaptation is so hard to prove. We clarified this point in the introduction (Line 89).

L75: The authors could clarify what is meant by “diverse environments”. Do the authors mean different environments (in different places), or variable environments (within a place)? While there is some empirical/modeling research suggesting that plasticity may help in colonizing new environments, in the long-term genetic adaptation is likely to occur (e.g. due to costs of plasticity). Within a location however, plasticity may be advantageous, but only if the cues are predictive of future conditions. I believe this is still an area of contention, and the authors should expand on these ideas to give a more balanced view of this field.

We have written this sentence, and cite specifically the seminal paper of Chevin and Lande, which describes the role of plasticity in climate change. The sentence now reads: “Such reactions may allow the organism to maintain high fitness when the environment becomes challenging, without having to evolve genetically [11].” (line 96)

Finally, I would suggest citing (and discussing) some works relevant to the current research, which are amiss. For example, there is a large body of earlier papers by Pigliucci & Schlichting on Arabidopsis plasticity, which could be discussed and cited and more recent works addressing challenges of mapping GxE (e.g. Sasaki et al 2015). Finally, I think a brief mention of some work done on experimental evolution in Drosophila addressing the polygenic nature of complex traits would also give some broader context for this work (e.g. works from the Schlötterer group generally, and from Thomas Turner and collaborators).

Plasticity and polygenic evolution are two topics that are both extremely timely and have a long history in evolutionary biology. As a result, it is practically impossible to do justice to all the great and inspiring work that we have read for this study. This comment, however, is very helpful to better identify the references that one should include.

1) regarding plasticity, we cite the work of Chevin and Lande, which we think is a landmark in the field (line 91), and now included the review by Pigliucci (2005) which provides a good summary of many concepts that Pigliucci prominently contributed to define (line 96), 2) mention Sasaki et al (line 469) to highlight the diversity of alleles that can contribute to the same phenotype in different environments and 3) conclude that polygenic adaptation is arguably the challenge of contemporary evolutionary genetics (conclusion, line 672).

**Results**

I’d like to start by making a rather lengthy technical comment on the statistical modelling of the growth data. I like how in Fig 1 there is an illustration of the type of model being fit and what the parameters of focus are. However, there is no sense of the uncertainty associated with these models, and I see this is an area where possibly a major improvement is required.

Firstly, a simple suggestion: it would be helpful if somewhere the authors displayed the underlying “raw” data as a line plot, with individual growth curves for each individual measured (this could be supplementary).

We agree with the reviewer that estimates may be poor if the fit to the growth curve is less good, however, we believe these would be conservative, since we actually focused our analyses on estimates of genotypic values, that incorporate three replicate measures of plant growth over time. In fact the logistic regression provides a very good fit to the data. It has been used before by the group of Olivier Loudet (see e.g. Marchadier et al. 2019).

To address this point, we have added the logistic regression plots of a representative set of genotypes as attachment to this letter (Suppl. Fig. S4).

For example, from the current Fig 1 it is not clear what parts of the curve are predicted (i.e. for which there are no data collected) and which are not. Crucially, there are no data for late days under HL, and LL only started to be measured at day 24. This should be more explicitly mentioned in the figure legend. As it is, it just seems we’re equally confident of what the curve looks like across the entire range of days.

We updated the legend to make this clearer. Note that more pictures were collected than was necessary for reconstructing the growth dynamics (especially at late time points, after the growth has plateaued).

The authors choice of using a logistic function seems to make sense for these data. However, could the authors detail what kind of logistic function was used: Three- Four- or Five-parameter function? And justify the choice made. Perhaps consider including the formula for the logistic model used in the methods (naming the parameters in the same way as they are used in the text, i.e.: “FS”, “t50”, “slope”).

It is a three parameter function since we assumed that rosette area was 0 on day 0 of the experiment. We now include this information in the methods (line 201)

In fact, I would encourage the authors to consider fitting the whole data in a single model. This could be done, for example, using a bayesian approach (using MCMC sampling) to simultaneously fit the logistic curve to all datapoints, while accounting for variation in the parameters of the model (FS, T50 and SL) due to accession, light, accession:light interaction and block. For example, the authors could consider using the brms R package and check their documentation on non-linear models (https://cran.r-project.org/web/packages/brms/vignettes/brms_nonlinear.html). For example, something like this for a 4-parameter logistic with the authors experimental design:

bf(SIZE ~ FS + (SS - FS) / (1 + (T / T50)^SL),

SS + FS + T50 + SL ~ light + (1|accession) + (light|accession) + (1|block),

nl = TRUE)

(please note I have not really tested this, I may be missing something here, and there may be different ways to parametrise the logistic equation that make more sense) Also, I would use a log-normal as the family to model the data, because the variance is likely normal on log-transformed data, but not on the original scale.

The advantage of fitting a model in this framework is that it immediately gives the authors all the parameters and their associated uncertainty in the posterior draws from the model. Based on this, the authors can then explore all the questions that they have, instead of fitting a myriad of GLMs on top of GLMs, Tukey tests, etc. For example, if I’m interested in GxE variation, then I can look at my posterior estimates for the sigma related to the GxE component (light|accession above). And I can visualise these as distributions, or mean +/- credible intervals, and so on.

I acknowledge that this might be challenging to implement. If that doesn’t work, at the very least, it would be better to fit a single mixed model to the logistic curve parameters, rather than several separate models as was done. Using R/lme4 package syntax, I think this would be suitable:

parameter ~ light + (1|accession) + (light|accession) + (1|block)

This is a “random slopes model” simultaneously accounting for accession, light treatment and block effects. Incidentally, this is also modelling the GxE component, and thus one can extract its variance. This would solve the authors perceived limitation that it is not possible to assess the variance of GxE. From this model the authors can get the relative magnitude of variances associated with genotype and GxE effects, which I felt were not clearly presented.

We are thankful to the reviewer for this interesting suggestion, and we tried to reanalyze our data using the Bayesian model provided by the reviewer.

However, in the end, we preferred to keep our original approach for four reasons:

First: our experimental design is nested at several levels. It is nested at level of the light treatment, because plants had to be grown in separate shelves, and at the level of genotypes, which have been individually monitored until plateauing at their final size. For this reason, we believe it is appropriate to estimate growth parameters for each genotype and controlling for block effects at that level. We further think it is more intuitive to most people interested in understanding the parameters that contribute to variation in growth dynamics.

Second: the way we quantified GxE variance follows exactly the logic proposed by the reviewer (Table 1). We have quantified the genetic variance associated with the interaction between genotype and treatment, but we note that this is not a correct estimation of heritability in the strict quantitative genetics sense.

Third: our treatment of plant growth is following previous publications by other groups (Marchadier et al. 2019),

Fourth: there is currently a consensus on linear models and their robustness to assumptions (see also **Schielzeth, H.**, et al. 2020. Robustness of linear mixed-effects models to violations of distributional assumptions. Methods in Ecology and Evolution [doi: 10.1111/2041-210X.13434](http://dx.doi.org/10.1111/2041-210X.13434)). This is not always the case for Bayesian analyses where the choice of prior distribution and their crucial impact remains intensively debatted. We have tried (really) hard to use the model recommended by the reviewer but it did not converge with simple priors.

To address this comment of the reviewer, we have improved the description of the experiment and the statistical analyses and now provide an R markdown.

**Section: “Ecological relevance of rosette growth variation”**

Apart from the modelling issues pointed above, the authors then conclude this section inferring that the variation in rosette size described is ecologically relevant. The argument being that measurements in growth chamber correlate with those in the field (on a single site). I was not fully convinced by this argument.

Here, there is another statistical point that needs clarity. Throughout the paper authors mention Pearson’s correlation and accompany it with a measure of r2 (r-squared). It would be good to clarify if the value the authors provide is indeed the squared Pearson’s correlation or not. Since Pearson’s correlation is usually denoted just as “r”, I assumed the values given are the squared pearson correlations. However in the legend of Fig 3 the authors write “pearson correlations (r2)”, which made me wonder exactly what the authors mean by r2 after all? The reason this is significant is that the squared Pearson’s correlation is interpretable as the proportion of variance explained by the relationship between two variables. And this impacts the interpretation many presented results.

For example, here the authors report r2 = 0.3 between diameter measured in growth chamber and field and use this as evidence that their trait measurements are ecologically relevant. If this is indeed the squared correlation coefficient, then the interpretation is that 30% of the variance in the data is explained by the relationship of these two variables. If, however, this is Pearson’s correlation then by squaring it we conclude that ~9% of the variance is explained by their relationship. These are substantially different conclusions.

Either way, for this particular case, I would say that given there is 70-90% unexplained variance, I would not take this as robust evidence to support the authors’ conclusion that the rosette growth measured in growth chambers is “ecologically relevant”.

We thank the reviewer for pointing out the incorrect mentions of r^2^. We corrected that in the manuscript (it is the Pearson's product-moment correlation).

Most studies of plant genetic variation measured in the lab omit to investigate whether the variation they report has any potential ecological relevance. Our study is one of the few that addresses this question. Given the polygenicity of the trait, given how field and chamber conditions differ, we actually rather share the opinion expressed by reviewers 1&2 that this relationship, even if obviously imperfect, is quite remarkable.

We have now mentioned this in the manuscript:

*This indicates that a significant part of the variation we report is ecologically relevant.* (line 399)

Also, it wasn’t clear to me which of the FS they are using for these correlations: on LL or HL? And what were the conditions in the field? Are they more similar to their LL or HL treatment?

We clarified this, it is FS measured in HL that correlates more similar to field (LL conditions are low compared to natural light at our latitude).

Minor comment: on L349 the term “lag” may be confusing, as there are models of growth that explicitly model a lag phase. T50 seems more correctly described as the inflection point.

We modified this.

**Section: “Environmental plasticity has the strongest impact on plant growth variation”**

I felt like this section was somewhat redundant with the previous one, in that it should be part of a fuller description of the phenotypic data and associated heritabilities. If the authors correctly model both G and GxE in a single model as discussed above, then they can discuss the relative magnitude of the genetic and non-genetic components of plasticity.

We apologize for the confusion. This aspect was quantified in a single model presented in Table 1, which we have now modified to highlight the significant but small proportion of variance explained by GxE. We have also clarified this aspect in the methods.

Furthermore, it would help to display the data as “reaction norm plots”, to better visualise the difference in responses at the accession level.

We feel there are too many genotypes for a graphic representation to be satisfactory. Note that this can be easily extracted from the genotypic mean that we provide for each accession, each environment and each parameter (Table S3).

Also, the way statistical “significance” is assessed in Fig 2 by running Tukey tests on the (average) outputs of the many models used is not correct, as it is not accounting for the fact these parameters were estimated with error. If the authors want to compare these groups, they should do so by setting appropriate contrasts within the model fit to the data (which again should preferably be a single model like discussed in my technical comment earlier). This also applies to Fig S6, S9 and possibly others.

The error term here is quantified by genetic variation within regions. Although we are aware there might be other ways to assess significance, we believe this level is appropriate for quantifying differences between regions.

**Section “Spanish genotypes show the most vigorous rosette growth”**

As discussed in my main points above, I find the discretisation of individuals into “regions” somewhat problematic. I would have preferred to see this information displayed either as correlations with PC1 and PC2, or simply shown on the map (effectively PC1 + PC2 seem to be capturing Latitude + Longitude, so they seem equivalent in a sense). This at least would somewhat avoid the possible artificial binning of the data as it currently is done, or at least help to see whether that binning is indeed justified. Again, I am not saying there is no difference across the range of Arabidopsis, but I’m not sure this analysis is helpful in assessing that.

As mentioned above, we have clarified this aspect in the methods section. Regions form metapopulations with distinct dynamics, based on previous reports of variation within and between populations (see e.g. Kronholm et al. 2010). They are discrete evolutionary units connected to different degrees by gene flow and time to their most common ancestor.

Ignoring the artificial discretization of data into regions, there is perhaps to much emphasis being put on p-values to interpret “significance”, when in fact it is perhaps the magnitude of the effect that we should focus on. For example, is a difference of ~1 day in t50 between Spanish and Northern European accessions on HL biologically significant (considering the average difference of ~10-20 days between LL and HN)? Maybe it is, I don’t know. . . But the authors should discuss this more fully. For example, is the bigger diameter a reflection of larger leaf surface area, or just longer petioles and/or leaves? In fact, are there other rosette traits that have changed substantially in response to light? This is not discussed anywhere, but it would be good to at least mention it. Even if the authors didn’t systematically collect these kind of data, just looking at the pictures and perhaps making some qualitative assessment to include in the discussion would be a nice addition.

We have opted for not speculating too much on effect sizes. A 1 day difference in t50 might well seem small, it nevertheless contributes to the larger final size that plants can reach, as well as a longer growth period. In particular, both t50 and FS indicate that Northern European plants are smaller. How this translates in terms of capacity to perform photosynthesis and invest into the root systems and later seed production is not something we can easily evaluate. While we acknowledge that this issue could be discussed in length, it seems to us that it is wiser to keep the manuscript as brief as possible. Therefore, we have only clarified that rosette diameter increase reflects an increased rosette leaf area (line 177). Note also that we have uploaded the raw images on DRYAD for future studies.

L382: The statistical treatment of flowering time is also problematic here, because the data is censored (many individuals never flowered) and thus the correlations with flowering time are misleading. The authors should not replace missing values with their maximum days of observation, and definitely not use standard tests in this context (where the variance between groups is clearly not homogeneous). If a statistical treatment is to be made, I think a survival model would be adequate. Or the authors could simply report the proportion of individuals that flowered over the course of the experiment.

We have simplified this paragraph and removed the discussion. The only point we wanted to make here is that the difference we see between Northern and Southern Europe are not due to differences in flowering time. We believe that this shows that our observation that rosette growth differences are regionally different (and potentially adaptive) is absolutely novel.

**Section “Chinese genotypes show that growth rate variation is constrained in evolution”**

I thought it was very interesting to include the Chinese accessions in the analysis, given their clear divergence from the rest. My conclusion from this, though, is that they are largely similar to the rest as far as growth dynamics is concerned. I could not follow how stabilising selection can be inferred from the data. If the trait is highly polygenic, there may be different genetic makeups that result in a similar phenotype. See, for example, the work of the Schlötterer group on drosophila. This, incidentally, migth be one of the reasons why the GWAS fails to identify associations.

There is no simple way of estimating stabilizing selection because we do not have information on the covariance with fitness. The reviewer, as we did, expected that Chinese genotypes could differ comparably more from the European genotypes. This is because these populations are genetically very divergent. Since it remains the same despite genetic divergence, it is a strong indication that the trait is constrained. We rewrote the sentence in the following way (line 433):

*“Despite a long history of population isolation that was magnified by a strong bottleneck after the last glacial period [38,71], the growth rate of Chinese genotypes was comparable to that shown by most European genotypes (Table S5, Fig. 2 a-d). Under LL conditions, Chinese genotypes showed lower t50 and FS values only when compared to Spanish genotypes (Table S5, Fig. 2 a-d). Under HL, genotypes from China did not differ significantly from those from any other region (Fig. 2, Fig. S11). The analysis of Chinese genotypes indicates that the phenotypic evolution of rosette growth does not scale with the extent of genetic divergence (Fst between Europe and China is 0.057 on average, with a standard deviation of 0.147, and much greater than Fst between Spain and Northern Europe, KS test, D=0.39, p<2.2e-16). Since growth rate has not significantly changed despite extensive population divergence, it appears to have been constrained by stabilizing selection in the Chinese lineage. “*

L415: the authors need to be careful in using the term “plasticity”, as here they seem to be referring to the genetic component of plasticity (GxE), or plasticity at the level of each accession. In fact, the labeling of Fig S6 left me slightly puzzled. What is the meaning of being above or below zero on this axis? This relates to my earlier comment that a fuller and clearer description of plasticity (in its genetic and non-genetic components) would be helpful. The y-axis of this plot should more clearly reflect what exactly is being displayed. A difference between which treatments? I inferred this may be the difference (LL - HL) of an accession relative to the overall average (LL - HL) across the whole population.

We have clarified this both in the text (line 412) and in the methods (line 210). We estimated genotype specific estimate of plasticity as a deviation of each genotype by the deviation of its response from the average response of the 270 genotypes included in our study. We clarified this, both in the legend of Fig. S6 (now S8) and in the method section.

**Section “GWAS reveal only two SNPs significantly associated with rosette growth variation”**

L427: the failure to detect associations might indeed be due to polygeny (which effectively translates as a small effect size for each causal locus), but also due to low frequencies of causal alleles and/or allelic heterogenity, all of which affect the statistical power. It would be good to present a more balanced interpretation of these results.

We mentioned this point (now line 469) but now rewrote this sentence to make it more clear. We added the mentioned constraints to the discussion .

L438-441: what is the minor allele frequency of these variants in the data? The fact that the variants picked have large effect size is perhaps not surpirising, but rather a statistical inevitability. If the SNP was above the threshold and has a low MAF, then the effect must be relatively large (it’s a statistical power issue). But there may well be many loci that are missed with equally low MAF, but effects too small to be detected here. The authors could consider adding a discussion of statistical power, as it is a part of the broader issue of genetically dissecting these complex traits.

We completely agree with the reviewer that diverse genetic set up can contribute to a polygenic architecture. Here is what we now write (line 469):

“Diverse genetic setups can result in such polygenic architecture: large effect size variants that are too rare to be detected, many variants with effect sizes too small to be individually significant, or the presence of multiple alleles at causal loci that will blur the genetic association signal “ and cite Sasaki et al. 2015 and Korte and Farlow 2013.

**Section: “Polygenic scores and functional enrichments confirm the polygenic basis of growth**

**variation”**

I like it how the authors made an attempt at further dissecting what clearly seems like a challenging trait with a complex genetic architecture. I do think this section is important, and really appreciate the extra insights one may get from exploring sub-significant associations.

We thank the reviewer for this positive feedback.

L447-455 seem to belong to the introduction, perhaps?

This would be indeed an option, but this sentence is there to help the reader follow our reasoning as we progress through the result/discussion section. Since the other reviewers did not point it out, we felt it was ok to leave this sentence as is.

L455 - the analysis on this paragraph could perhaps be improved by providing some level of uncertainty. The correlation between predicted and observed is relatively low. What kind of correlation does one get if picking ~20 random SNPs with similar allele frequencies as the ones < 10e-4? The authors could perhaps run this a few hundred times and provide with the distribution thus obtained. The authors conclude this paragraph with a reference to Table S11, which I felt needed more explanation. I confess not being familiar with the details of the P_Qx statistic, but even so felt that there could be a better explanation of these results.

We have remodeled this paragraph quite a lot (see above), in order to show that the polygenic scores are imperfect predictors of growth variation.

The authors could perhaps also consider fitting a polygenic model using some form of regularised regression (such as ridge regression), to get estimates of snp effects across the genome. Presumably this may still be influenced by population structure (although the all-SNP model should somewhat take it into account), but at least it would be another complementary analysis to assess the polygenic aspect of the trait. See for example the work of Kooke et al (2016) https://doi.org/10.1104/pp.15.00997.

We believe that if the focus of the paper were to describe a complex trait, it would be important to use multiple methods. But here, our focus is to understand whether growth rate varies between regions of the area of distribution of the species and evaluate whether this variation may be adaptive.

It could also be worth having a fuller discussion of existing work on the polygenic nature of growth-related traits in plants (specifically in crops, where there is often higher statistical power due to the use of RIL populations).

This is an interesting aspect. For brevity, we opted for citing the review on polygenic evolution by Wellenreuther and Hansson. Note that we are citing important manuscripts such as Berg et al. 2019, that provide state-of-the-art discussions of the challenges faced by studies of polygenic evolution.

L497: High Fst is not synonymous with adaptation. There are several scenarios that may lead to high Fst, some of which have nothing to do with selection. There has been ample discussion about this in the field of population genomics of the last decades (e.g. the recent review of Wolf & Ellegren 2017). The authors should be more careful in making evolutionary interpretations based on Fst.

This is true, but our work is not on searching for outlier Fst per se. We investigate how outlier Fst associate with GWAS hits, two observations that are independent (if population structure is corrected for). Our extensive discussion of the importance of the population structure that remains even after correction is, in our view, what expresses our careful interpretation.

It is also not clear between which populations Fst is being calculated (this is only mentioned in the methods), and on which basis those populations are chosen. As I already raised a major concern in splitting populations in these “regions”, I am not sure how to interpret this analysis of Fst. At the very least, it would be good to see what the Fst profile is across the genome, as it is unlikely to be homogenous.

We have clarified this accordingly in the text.

**Section: “No association between per-individual burden and growth”**

I did like the approach of looking at loss-of-function alleles. However, I was confused as to why these variants were not already included in the GWAS dataset? Or at least SNPs linked to them. Perhaps the authors could explain this a bit better, to understand why they had to run separate association scans for the two analysis.

We improved this aspect in the corresponding result section (line 572). Loss-of-function variation is coded as 0 or 1, but different alleles can cause the loss of function. In other terms, a single LOF variant can correspond to several SNPs (and account for problems caused by allelic heterogeneity). So these datasets are different in nature.

Again, I am not convinced about the evolutionary/ecological interpretations of the results. The “Northern” group of accessions that the authors use to test an hypothesis about edge populations, actually occupies a very broad geographic area. Of course the definition of a “population” is to a certain extent arbitrary, but I think that a collection of accessions spanning an area of many (thousands?) of km2 is too broad to address these kinds of questions. I think they would need to be addressed with sampling at a more local scale, and so the current collection of accessions does not seem suitable for such population genetics questions.

We believe the reviewer underestimates the time scale at which many evolutionary processes can leave their signatures. As mentioned above, the regions form metapopulations that can clearly be distinguished by their evolutionary history. In a population genetics sense, a whole species forms a population.

At a more technical level, I also wonder if the count of LOF alleles needs to be weighted by the number of synonymous mutations? In particular, the increased number LOF alleles in Chinese accessions, is it accompanied by a general higher number of variants in the coding regions?

No, this should exactly not be done. See Simons and Sella 2016, which we cite in our manuscript. The reason for this is that a load of deleterious mutations is something different than a bottleneck. A bottleneck increases the ratio of non synonymous to synonymous variants, but this is because after a bottleneck, there are more non synonymous variants that have no effect on fitness. Simons and Sella do a great job at carefully explaining these processes.

**Section: “FS variation might reflect local adaptation at the regional scale”**

Again, I am not sure how to interpret these results. It is not clear how strong the signals reported by the authors is. For example, the analysis of Fst is simply reported with a p-value from a KS test. But given the high number of data points (1360 SNPs), it’s unsurprising that the authors get a low p-value, no matter what test they do. As before, what is the Fst profile across the genome, and what patterns are observed around these supposedly selected SNPs? If these were selected in the past, are there signatures of a selective sweep? If not, what are the models proposed for selection? Were these soft sweeps, or hard sweeps that happened so long ago as to preclude any signals from being found in the genome (how does that fit with the timing of these colonisations)?

We apologize for the confusion. The comparison that was tested was not properly reported. We compared the Fst distribution of associated SNPs, with the distribution shown by random samples of an equal number of SNP. We rewrote this sentence (line 615):

“Compared to the Fst distribution of 10 000 random sets of SNP, the Fst distribution of the 1360 SNPs associated with any of the growth parameters at *p*<10^-4^ was significantly shifted towards higher values (p<10^-4^).”

I suppose my concern is that these are complex questions for which the current set of accessions is perhaps not suitable to answer. I would advise the authors to put less emphasis on population-level evolutionary questions and present a fuller description of the trait variation and the difficulties in uncovering the genetic architecture of such traits.

We did not look for sweep signatures around these regions. The reason for this is that the exact demographic history of these populations is very hard to model. Sweep detection methods like SweeD or others would not be very effective. Polygenic adaptation actually seldom creates significant sweep signatures (either soft or hard). For brevity, and given that we focus more on the phenotypic signatures of selection rather than the molecular one, we prefer not to mention the soft/hard sweep debate.

**Data availability**

Given the very heavy analysis-driven nature of this work, analysis code should be made available to allow interested researchers (and reviewers!) understand the technical analysis implementations used by the authors.

(This could have helped me, for example, to understand what the authors meant by Pearson’s correlation and their “r2” notation, or their many models’ specifications, which I often struggled to understand).

We agree with the reviewer. We now provide an R markdown file of the analyses.

The authors only provide processed data, but not the primary raw data (photographs). Making this available seems like an invaluable contribution to the community.

Raw image data and image analysis scripts is being stored in the DRYAD repository (doi:10.5061/dryad.s1rn8pk5m, early access before publication: <https://datadryad.org/stash/share/7eqIIcdI4ZeC8rgDjfXyXvx1h3srkXPBNMvnQtlee7s>).

It would be good to mention sample sizes in figure legends.

Modified accordingly

Table S11: in the legend, what do the authors mean by the “second half of the table”?

Thank you for pointing out this typo. We have corrected it.

From Table S2 (supplementary data file), only 5 individuals have measurements for days 4 and 15 (belonging to two genotypes). Also, individuals from LL treatment are only measured from day 24 onwards. I guess these issues may have to do with logistic constraints that are natural to such large experiments. However, these should be mentioned in the methods, along with any reasons (biological, logistical, etc.) that led to the collected data.

We have clarified in the methods that when needed, in case the fit to the logistic curves failed, we measured rosette diameter at additional time points (line 169). We are therefore confident that differences in growth dynamics are not due to differences in the fit to the logistic curve.

L140 authors mention plants photographed approximately bi-weekly. Later in the paragraph they say measures were done weekly. This seems inconsistent. (data in Table S2 does suggest approximately weekly measurements).

See the point above, we have more pictures than measurements and only measured once per week.

For the climate data, PCA was used to summarise the variables. The authors should be more explicit that only the first 2 PCs were retained and perhaps mention how much variance was explained by them. It should also be clarified if the data was scaled prior to running the PCA (I guess it should, since these variables are measured in very different units/scales). Also, all of the individuals from “Western Europe” seem to have missing values for PC scores. Why is this?

We now provide the variance explained (legend of Fig. S23) and a table with loadings (Table S11).

Some of the references are duplicated at the end of the document. There is also some mis-referenced work -I spotted one reference “51”, when I think it should be “53”. The authors should carefully revise all their references.

We apologize for this problem caused by our reference manager when generating the last version of the manuscript. We unfortunately did not notice it. We have paid specific attention to the problem this time.

Some of the figure legends don’t provide measurement units (specifically those relating to “GxE” and “slope”).

We thank the reviewer for pointing this out. Slope has no unit because slope is a multiplicative factor, but GxE has the same unit as diameter measurements (cm). We now clarify this in the methods section (line 203 & 212).
