# Supplementary material for: Polygenic adaptation of rosette growth in Arabidopsis thaliana
Source: PLoS Genet. 2021 Jan 25;17(1):e1008748. doi: 10.1371/journal.pgen.1008748 (PMC7861555; doi:10.1371/journal.pgen.1008748)
Supplement: Attachment — Submitted filename: response_reviewer_minorBW2.docx [file pgen.1008748.s037.docx]

**Detailed Response to Editor and Reviewers.**

**Editor**

Thanks for you patience with this very long review process, which was mostly a Covid-causalty. For what it's worth, the manuscript has clearly improved greatly as result of revision!

As you will see from the reviews below, one reviewer is still unhappy with the manuscript, and does not think you have addressed his/her concerns. The points raised are valid, but the discussion is getting rather philosophical, and I think it would be wrong to hold up the paper over something like this. The paper is clearly written, and readers can judge for themselves. However, as you address the other minor comments raised below, please consider changing what you say slightly to accommodate the fact that there are (non-crazy) people who are not convinced by your interpretation of polygenic scores and GO enrichment.

We thank the editor for his appreciation of our work. We indeed tried to write a paper that is honest with some of the limitations that we currently have, when analyzing polygenic patterns of adaptation. Regarding the validation of the polygenic basis of our trait, however, we think the reviewer has not totally realized that we did contrast our polygenic scores and GO enrichments to random expectations. Indeed, we did verify that polygenic scores are better predictors when computed from real data than from permuted datasets, and that GO enrichments were selected on a p-value threshold that was lower than what we may observe with random datasets. So these functional enrichments are real. In addition, by comparing functional enrichments among sub-significant GWAS hits and among higher Fst SNPs, we made sure that these enrichments are not reflecting the effect of outlier variants independent of the phenotype.

To make this clearer, we modified the text in the methods (line 312 and 331) and result (line 547 to 564) sections.

We hope that these modifications will convince you that we have been very cautious in our effort to validate the collective signal given by sub-significant GWAS associations. We have added all the minor modifications listed by the reviewers and hope that you will judge our manuscript worthy of being published in Plos Genetics.

**Response to comments by reviewers**

**Reviewer #1:** The revised manuscript by Wieters et al, contains considerable changes which present the conducted research in a clear and understandable way. I also want to thank the authors for clearly answering each of the reviewers' comments.
I agree with all the answers to the comments and with the changes that were made to the manuscript.
Congratulations to the authors with this very nice work.

Thanks for the appreciation!

**Reviewer #2:** The authors did a great job of streamlining and clarifying the manuscript; both the analyses and the narrative of the manuscript are much easier to follow in this revised version, and this clarified some of the points I found confusing in the original manuscript. Polygenic traits are messy and I really appreciate how thorough the authors are in their approach to this study.
However, some of the concerns I raised in the first round of reviews still stand. As a consequence, I am not convinced that the data, as they are presented, fully supports all of the main conclusions put forward by the authors. Below I am reiterating the points that I think were not fully addressed by the authors.

- In my original comments I had discussed the issues I had with the way polygenic scores results were validated and the interpretation of the GO analyses. I appreciate that the authors have been more cautious in reporting the results of the GO analyses. However, the analyses are largely unchanged, and so is their interpretation (the corresponding section of the manuscript is still titled “Polygenic scores and functional enrichments confirm the polygenic basis of growth variation”, as is, by and large, the way they are reported in the abstract).

As it stands, the polygenic scores results look promising but the way they are being validated (by using two replicates to predict phenotypic values for the third) does not look convincing to me. Both I and reviewer 3 suggested that comparing those results (Table S7) to a distribution based on random SNP sets could solve the problem.

As mentioned in my original comments, the one polygenic score calculation for a set of 27 random SNPs presented in Table S8 does not seem very convincing (and I still do not think it can be fully compared to the results in Table S7). Unfortunately, but as the authors themselves point out perhaps not surprisingly, the attempt to predict phenotypic values for a subset of accessions based on polygenic scores was not successful.

The question is not whether a random set of SNPs would give the same results (it clearly doesn’t, we show it now also in Table S7B) but whether population structure alone could give this result. Since the predictive power of random SNPs is much lower, we conclude that there is some true genetic signal in our sub-significant GWAS associations. **To clarify this aspect, the text was rewritten** (see line 312, 331 and 547-564).

I concur with the authors that the definition of GO categories is limited by the availability of experimental evidence for gene functions. While in my mind this makes interpreting results from GO analyses even harder (if we cannot trust the categories, why look at them in the first place), looking at it from this perspective could be interesting if there was independent evidence that the GWAS signal really describes the polygenic basis of those traits (i.e., “I am sure that those associations are meaningful, let’s see if I can learn something about them by looking at GO categories”).

However, in this case GO categories are used to validate the goodness of the GWAS results, which I find less convincing (i.e., “since there are significantly enriched GO categories, and some of them could be linked to the traits we are studying, it means that sub-significant SNPs accurately describe the polygenic basis of those traits”). As stated before, the fact that there is no overlap between enriched GO categories for GWAS associations with p < 10-4 (Table S9) and ranked GWAS associations (Table S10), makes me even less confident about the interpretation of these results.

Neither this analysis, not the polygenic score results, seem to definitely confirm that the sub-significant GWAS associations describe the polygenic basis of growth variation.

Here, we don’t entirely follow the reasoning of the reviewer. That we cannot interpret how some of the enriched categories may affect growth does not mean that the enrichments are not significant. In fact, whether you trust the significance of the enrichments MUST be independent of whether they make immediate sense. To trust the enrichment, we called them significant at a p-value that is lower than enrichment based on a comparable set of SNPs. In addition, we removed tandem duplicates from the annotated set, to make sure that significance is not inflated by one SNP mapping in the middle of a multigenic family (actually a problem that tends to be overlooked in many studies of this kind). And finally, we have verified that these enrichments do not overlap with those given by population structure outliers.

We have clarified this both in the methods and result sections (line 331-346 and lines 547, 551 and 643).
- Likewise, I am still unconvinced that stabilizing selection is necessarily the explanation for the lack of phenotypic differentiation between Chinese and European (one of the other main results reported in the abstract), instead of, for example, physiological limitations or convergent adaptation. While there is no harm in proposing stabilizing selection as a possible reason for the observed pattern, this particular explanation is reported several times throughout the manuscript.

We tried to clarify this better and now write on line 447 (results section): “A parsimonious explanation to the fact that growth rate has not significantly changed despite extensive population divergence, is that the evolution of growth rate is likely to be constrained by stabilizing selection around a growth optimum [1]. ” and cite Mitchell-Olds (1996).

- In response to one of my comments, the authors added to the abstract and elsewhere that the light conditions they used mimicked latitudinal differences in light intensity, which is useful in better framing the scope of the study. However, it is difficult to compare the light intensity reported for the growth chambers (in µmol m-2 s-1, probably of PAR) to the total radiation reported in Figure S3 (kJ m-2 day-1, which I guess is total radiation), especially not knowing the exact wavelength distribution in the growth chambers. It would be helpful to express those values in a format that is more comparable, and explain more in details how the HL and LL are comparable to latitudinal differences; light intensity in HL is more than double that in LL, while, according to Figure S3, average radiation in Spain is only ~1.5 that of North Europe.

We are afraid that on this matter, our experiment is “mimicking” differences in light intensity, rather than “reproducing the natural differences in light intensities experienced by *A. thaliana* population in Northern Europe vs. Spain”.

To be even more transparent about this limitation, we added the light spectrum of the chamber to fig. S3, which also features the quantitative variation in light intensity experienced in Spain vs. Northern Europe. Obviously, light intensity in the chamber is not as strong as in natural conditions. In addition, a direct comparison, with LEDs is difficult, because LEDs do not have the green spectrum.

- As other reviewers noticed as well, the division in four groups is not always well justified. While I am convinced about the groupings for Spain, Northern Europe and China (based on the PCA in Fig S2), Western Europe does not seem to form a group of its own in that analysis.
From a population genetics perspective this group is at the end of a continuum but also belongs to a geographical area that is intermediate between Spain and Northern Europe. The number of genotypes in this group is however small and it had to be excluded from the major analysis. We decided however to leave things as they are in the manuscript, because considerations on this group of genotypes are not important for the conclusion of our manuscript, but by increasing the number of phenotyped accessions, it increased the power of GWAS.
As an aside, I realize there was a typo in my previous comments; in point 5), I meant to write that heritability for SL and t50 ranges between 0.07 and 0.2, not 0.02 – sorry if that generated any confusion.

No problem.

Reviewer #3:In their revised manuscript, the authors amend and clarify several points raised by myself and the other reviewers. Although there haven't been major changes in the content, structure and conclusions of the manuscript, the many changes done throughout seemed to me to clarify the diverse and rich set of analysis done.

The authors' replies and counter-arguments to some of my comments did increase my appreciation for their approach of looking at the results of this garden experiment seen from an evolutionary lens (rather than a purely quantitative genetics perspective). Apart from a few remaining comments below, I think the manuscript as is provides enough clarity to allow readers to draw their own conclusions from the interesting correlations found between rosette growth variation and patterns of genomic variation seen across different subsets of these accessions.

We thank the reviewer for his appreciation of our efforts.

I thank the authors for substantially clarifying the statistical treatment of their trait data (and providing with analysis code). This has really helped me understand their approach and better interpret their figures. I was sorry that my suggestions for alternative analysis didn't prove fruitful, I'm sorry for the time you might have spent on this without good returns. My main intention was to gain an understanding of the uncertainty associated with the growth curves. The authors provide a new supplementary figure S4 with individual curves, which goes some way at communicating this.

We thank the reviewer for his suggestion and indication of how to apply a Bayesian model to our data. We are also testing these models for other dataset we have in the lab. So definitely, this suggestion was very interesting to us, even if it did not help describe better the dataset presented in this manuscript.

I have a few remaining questions, which the authors may wish to address.

L148: very few points for Western Europe are visible in Fig S2, and the few that are visible seem to occur mixed in with Spanish and Northern European accessions (reviewer #2 also highlighted this). Perhaps adding transparency to the points would help visualise them? Also, would it be possible to add number of accessions in each group to the figure legend? Am I correct in understanding that these groupings are based on the "admixture group" provided by the 1001genomes project: http://1001genomes.org/accessions.html? If so, it may be worth explicitly stating so, as this would have clarified my previous concerns about the discretisation of these data (also mentioned by reviewer #1 and #2). I think this is implicitly mentioned in Table S1 legend, but might be worth having it more visibly here in the methods.

Note that the number of genotypes in each group was already given in the legend of Fig. 2 and Fig. S8. The grouping was made on the basis of geography and genetics. We clarified this on line 149.

L152/L197: The description of the experiment as following a "nested design" might be confusing as some of the factors are crossed and others nested. If I understood the design correctly, I think it is technically a split-plot design (https://besjournals.onlinelibrary.wiley.com/doi/full/10.1111/j.2041-210x.2012.00251.x). Within each treatment the experiment was blocked three times (blocks nested within treatment) and all accessions occurred in each of the 6 blocks (accessions and blocks are crossed). Accession and light treatment are therefore indirectly crossed (i.e. all accession-treatment combinations were done - this is what allowed looking at GxE interaction).
Agree, this is the correct description. We modified the text accordingly.

L169/L428: "For genotypes without a flowering individual by the end of the experiment, a flowering time value of 59 or 90 days was assigned to HL and LL plants" As I mentioned before, this is not a correct treatment for censored data. The average (and Tukey test) as calculated by the authors is problematic as it is biased by the number of non-flowering individuals in each group. For displaying these data in Fig S10 the authors could consider plotting those individuals that did not flower as points with a different shape at the upper edge of the y-axis (to clearly indicate they are censored data) and not include them in the boxplot calculation. The authors could report the percentage of individuals that flowered for each population. As the claim is that there are no substantial differences between NE and SP it would be useful to see that there is no substantial differences in the proportion of individuals that flowered in this experiment.
Here, the question is whether early flowering in one region could in fact be the reason that rosettes are smaller. Since this analysis answers this question, we prefer to leave this as is.

Line 218 "as the ratio of." didn't finish the sentence.
Fixed, thanks for pointing it to us.

L218: "The heritability of GxE could not be estimated..." As I mentioned, I believe GxE variance could be estimated by fitting a random slopes model, if the authors wish to do so, something like: parameter ~ treatment + (treatment|accession) + (1|treatment:block)
Otherwise, it may be best to omit this statement from the methods.
We know GxE is heritable, because we have a significant interaction between treatment and accession, but we do not know how to compute heritability in a way that is comparable with the heritability measured for phenotypes we scored in three independent replicates.

We write in the methods (line 216):

“Briefly, genetic and environmental variances were estimated using the *lme* function from the *nlme* package [45], with the block as fixed and genotype as random effect and heritability was determined as the ratio of genetic variance over the total variance. The heritability of GxE was not estimated, because we quantified plasticity on the basis of changes in genotypic means in the two light conditions.”

L432: I still could not follow the argument in favour of stabilising selection (also raised by reviewer #2). The high Fst is a consequence of the separation of these lineages (with the bottleneck further increasing their relative divergence - as within-population diversity must have dropped in Chinese populations). But why would that be expected to result in a change in rosette growth? What level of phenotypic divergence (in a polygenic trait) would be expected to accompany a change in relative genetic divergence? Are other traits in Chinese accessions significantly different from European accessions such that a lack of difference in rosette growth is surprising? Are the data also compatible with stabilising selection across the whole range of Arabidopsis (not just Chinese populations) or evolution around a trait optimum, e.g. due to physiological constraints as reviewer #2 mentioned? Without data on fitness disadvantage of extreme phenotypes, it seems hard to infer whether stabilising selection is at play in this system.

As mentioned, we are surprised by how controversial this statement has turned out although it seemed both simple and parsimonious to us. In the absence of constraints, Qst = f(time), because alleles underpinning its variation drift. So in the absence of constraints, we expect Qst china/spain > Qst Spain, Norway, because the divergence spain/china is older than the divergence Spain/Norway.

We agree that other things could have happened, but stabilizing selection seems the most parsimonious. We now write in line 447: “A parsimonious explanation to the fact that growth rate has not significantly changed despite extensive population divergence, is that the evolution of growth rate is likely to be constrained by stabilizing selection around a growth optimum [1]. ” and cite Mitchell-Olds (1996).

L496: "The associated sets were composed of 22 to 37 unlinked SNPs". Is there any overlap in the SNPs for each trait?
This refers to the set of SNPs that were pruned from linked SNPs, so it is not so relevant to determine the overlap. Some traits do associate with common regions in the genome, especially if they are correlated, but rarely with the exact same SNP. We did not include this because we felt that describing the overlap would be lengthy and not help make the results clearer.

L576: It might be worth mentioning that a common association for T50-LL and T50-GxE is not surprising given the high correlation between these traits (Fig S18). In fact, they are not independent traits, as T50-GxE is calculated from T50-LL and T50-HL: possibly there's lower variation in T50-HL, and therefore variation in T50-GxE is mostly due to variation in T50-LL.

Yes, T50 plasticity is the variance in T50 LL that is independent of the T50 variation in HL. So that the loss of function allele associates with both is telling us that this association is dependent on the growth environment.

Fig S22: have the authors corrected these tests for multiple testing?
No. But does it make sense to correct for multiple testing in this analysis of polygenic scores when we have shown that the traits themselves are not independent, even after correcting for multiple testing (Fig. S18).

L669: the authors nicely caution that Fst and polygenic scores might be confounded by structure. I was wondering if there is a relationship between Fst and SNP effects (at all SNPs, not just p < 1e-4)? As the authors discuss (L489), it is challenging to disentangle variation that is confounded by structure from variation that is not (despite the statistical adjustment with kinship matrix). I was wondering if looking at this relationship would help in any way to investigate the extent of this issue in these data?

We believe that this comments is addressed in fig. S19, which shows that high Fst alleles are enriched in GO categories that are distinct from those enriched in GWAS.

L714: "(blue line)" might be meant as "dashed line".

Thanks for picking this up. We corrected this typo.

L756/L762: check numbers of referenced figures

Yes, there is a mistake there. The legends in the supplement is the correct one. We corrected it in the main manuscript.
